# CopM is a novel copper-binding protein involved in copper resistance in *Synechocystis* sp. PCC 6803

**DOI:** 10.1002/mbo3.231

**Published:** 2014-12-26

**Authors:** Joaquín Giner-Lamia, Luis López-Maury, Francisco J Florencio

**Affiliations:** Instituto de Bioquímica Vegetal y Fotosíntesis, Universidad de Sevilla-CSICAmérico Vespucio 49, E-41092, Sevilla, Spain

**Keywords:** Copper, cyanobacteria, metal homeostasis, plastocyanin, protein export

## Abstract

Copper resistance system in the cyanobacterium *Synechocystis* sp. PCC 6803 comprises two operons, *copMRS* and *copBAC*, which are expressed in response to copper in the media. *copBAC* codes for a heavy-metal efflux–resistance nodulation and division (HME-RND) system, while *copMRS* codes for a protein of unknown function, CopM, and a two-component system CopRS, which controls the expression of these two operons. Here, we report that CopM is a periplasmic protein able to bind Cu(I) with high affinity (*K*_D_ ∼3 × 10^−16^). Mutants lacking *copM* showed a sensitive copper phenotype similar to mutants affected in *copB*, but lower than mutants of the two-component system CopRS, suggesting that CopBAC and CopM constitute two independent resistance mechanisms. Moreover, constitutive expression of *copM* is able to partially suppress the copper sensitivity of the *copR* mutant strain, pointing out that CopM *per se* is able to confer copper resistance. Furthermore, constitutive expression of *copM* was able to reduce total cellular copper content of the *copR* mutant to the levels determined in the wild-type (WT) strain. Finally, CopM was localized not only in the periplasm but also in the extracellular space, suggesting that CopM can also prevent copper accumulation probably by direct copper binding outside the cell.

## Introduction

The transition metal copper is an essential element for almost, if not, all organisms on earth. The metal-based biochemical reactions have been selected and conserved during the evolution of life, because the chemical properties of these elements make them suitable as structural and active cofactors in enzymes. In this sense, copper is required for essential biological processes such as energy generation, iron uptake, and protection against oxidative stress. The ability of copper to donate and accept electrons alternating between its cuprous Cu(I) and cupric Cu(II) oxidation states make it an excellent cofactor in enzymes. However, the same redox properties that are exploited by metalloproteins turn it into a toxic agent. When copper is in excess, it can generate reactive oxygen species (ROS) through Fenton-like reactions, destabilize Fe-S clusters, and compete for the binding sites of other metalloproteins (Macomber and Imlay 2009; Robinson and Winge 2010). Hence, the amount of copper ions inside the cell must be tightly regulated to ensure that copper gets delivered to every copper-containing protein and to prevent spurious copper binding to other metalloproteins (Waldron and Robinson 2009; Waldron et al. 2009; Robinson and Winge 2010).

Copper homeostasis is a complex process involving efflux, sequestration, and oxidation of this metal ion. In bacteria, active efflux is one of the key mechanisms for copper resistance and it is mediated mainly through the P-type ATPases, such as *Escherichia coli* CopA (Rensing et al. 2000; Grass and Rensing 2001b; Rensing and Grass 2003), that transport actively Cu(I) from cytosol to periplasm and heavy-metal efflux–resistance nodulation and division (HME-RND) system, like CusBAC (Grass and Rensing 2001b), that are able to export Cu(I) from both cytosol and periplasm to outside the cell (Kim et al. 2011). Periplasmic copper metabolism has an important role in copper homeostasis, not only because all known copper-dependent proteins in Gram-negative bacteria are located in either the periplasm or the cytoplasmic membrane but also because it is the main barrier to avoid copper entry to the cytosol. Copper can be reduced in the periplasm by either specific periplasmic proteins or unspecific oxidation of cysteines (Depuydt et al. 2009). Since Cu(I) can lead to generation of ROS in the periplasmic space and this is the main ion form taken up by the cell, mechanisms to detoxify Cu(I) in this compartment are necessary. To achieve this, copper homeostasis systems usually contain periplasmic copper-binding proteins such as CusF in *E. coli* (Franke et al. 2003; Loftin et al. 2005), CueP in *Salmonella typhimurium* (Pontel and Soncini 2009), CopK in *Cupriavidus metallidurans* (Monchy et al. 2006), and in some cases, multicopper oxidases (MCO) such as CueO or CuiD (Grass and Rensing 2001a; Espariz et al. 2007; Achard et al. 2010). In *E. coli*, when Cu(I) reaches high levels, CueO oxidizes Cu(I) to Cu(II) protecting periplasmic proteins and diminishing Cu uptake into the cytoplasm (Grass and Rensing 2001a; Singh et al. 2004). The expression of genes encoding copper resistance elements acting in the periplasm is mainly under the control of two-component systems that directly detect periplasmic copper levels, of which the best characterized member is CusRS in *E. coli* (Mills et al. 1993; Munson et al. 2000; Osman and Cavet 2008; Zhang and Rainey 2008). CusRS controls the transcription in response to copper of the *cus* operon, *cusCFBA* (Munson et al. 2000), which encoded for a HME-RND efflux pump, CusCBA, and the small periplasmic metallochaperone, CusF, that together with CusB delivers the toxic ion from the periplasm to the extracellular media (Bagai et al. 2008; Mealman et al. 2011). Additionally, it has been found in *S. enterica* serovar Typhimurium and other bacteria that do not contain the *cus* system another CueR-regulated gene, *cueP*, which encoded for a periplasmic protein involved in copper resistance (Pontel and Soncini 2009). A *cueP*-deleted strain of *S. typhimurium* was found to be highly susceptible to copper, especially under anaerobic conditions. Recently, it has also been reported that CueP can supply the copper ion to the periplasmic Cu,Zn-superoxide dismutase (*SodC*II; Osman et al. 2010, 2013).

Cyanobacteria are an attractive model to investigate the systems implicated in copper homeostasis since they have an internal copper requirement for two proteins: the blue copper protein plastocyanin and the caa_3_-type cytochrome oxidase. These two proteins are localized into a special internal membranous structure, the thylakoids, where photosynthesis and respiration take place in cyanobacteria. In cyanobacteria, copper metabolism has been mainly analyzed in *Synechocystis* sp. PCC 6803 (hereafter *Synechocystis*). Copper is imported into the thylakoids by two P_I-_type ATPases, CtaA, and PacS (Tottey et al. 2001, 2002). While CtaA imports copper from the periplasm to the cytosol, PacS transports it to the thylakoid lumen. These two transporters are assisted by SynAtx1, a copper metallochaperone, that interacts with the aminoterminal domains of both ATPases ensuring copper is transported to thylakoids and avoiding the presence of free copper into the cytosol. Furthermore, glutathione has been shown to cooperate with SynAtx1 to buffer cytoplasmic copper levels, preventing deleterious side reactions (Tottey et al. 2012). Recently, we have described a copper resistance system in *Synechocystis* that comprises a two-component system, CopRS, an HME-RND export system, CopBAC, and a protein of unknown function, CopM (Giner-Lamia et al. 2012). These proteins are encoded by two operons: *copMRS* (of which two copies exist: one in the chromosome and the other in one the endogenous *Synechocystis'* plasmid, pSYSX) and *copBAC*, which is only present in the plasmid pSYSX. Expression of both operons is regulated by CopRS in response to copper in the media and *copM* and *copR* are the most induced genes by copper (Giner-Lamia et al. 2012, 2014). Mutants in either *copRS* or the *copBAC* render cells more sensitive to copper and accumulate higher amounts of copper than the wild type (WT; Giner-Lamia et al. 2012). In contrast, little is known about copper homeostasis in the periplasm beyond that the most abundant Cu(II)-binding periplasmic protein in *Synechocystis* is CucA (copper cuprin A), a quercetin 2,3-dioxygenase (Tottey et al. 2008), and the iron-binding protein FutA2 (Waldron et al. 2007). Furthermore, FutA2, an Fe(III) periplasmic-binding protein, change its mobility after bathocuproinedisulfonic acid (BCSA, a copper chelator) treatment and deletion of *futA2* leads to lower copper-dependent cytochrome oxidase activity in the plasma membrane together with copper hyperaccumulation in the periplasm (Waldron et al. 2007). These data suggest that FutA2 affects copper uptake into the cytosol (Waldron et al. 2007).

In the present work, we present evidence that CopM is a periplasmic/extracelullar protein involved in copper resistance in *Synechocystis*. Deletion of *copM* generates a strain (ΔΔ3RS strain) that is as copper sensitive as the strain lacking the *copB* gene (COPB strain), but less than a mutant strain lacking the *copRS* system (ΔΔ3 or COPR strains). We have also determined that CopM is a protein able to bind Ni and Cu. Although CopM was able to bind both Cu(I) and Cu(II) with a 1:1 molar ratio, it showed a higher affinity toward Cu(I) with a dissociation constant *K*_D_ 3.7 ± 0.4 × 10^−16^ mol/L. Additionally, constitutive *copM* expression in a COPR strain leads to an increase in copper resistance. This expression also reduced the elevated copper and plastocyanin levels observed in the COPR strain compared to the WT strain levels. These suggest that CopM is able to reduce copper content in *Synechocystis*. Moreover, we have also shown that an important fraction of CopM (∼30%) was found in the extracellular space, which suggests that CopM is able to sequester copper in the periplasm and/or extracellular space avoiding copper accumulation.

## Experimental Procedures

### Strains and culture conditions

*Synechocystis* and *Anabaena* sp. PCC 7120 strains used in this work are listed in Table1. All *Synechocystis* strain cells used in this work were grown photoautotrophically on BG11C-Cu (lacking CuSO_4_) medium (Rippka et al. 1979) at 30°C under continuous illumination (50 *μ*E m^−2^ sec^−1^) and bubbled with a stream of 1% (v/v) CO_2_ in air. For plate cultures, media were supplemented with 1% (wt/vol) agar. Kanamycin, nourseothricin, chloramphenicol, and spectinomycin were added to a final concentration of 50, 50, 20, and 5 *μ*g mL^−1^, respectively. Experiments were performed using cultures from the mid-logarithmic phase (3–4 *μ*g chlorophyll mL^−1^) in BG11C-Cu medium supplemented with different amounts of CuSO_4_ and NiSO_4_, when required.

**Table 1:** Cyanobacteria strains used in this work

Strain	Relevant genotype	ORFs	Source
WT	*Synechocystis* sp. PCC 6803	–	Laboratory collection
*Anabaena*	*Anabaena* sp. PCC 7120	–	Laboratory collection
COPR	*ΔcopMRS::SpΩ pcopR::C.C1*	*slr6040*, *sll0788*, *sll0789*, *sll0790*	(Giner-Lamia et al. 2012)
PCOP	*ΔpcopR::C.K1*	*slr6039*, *slr6040*, *slr6041*	(Giner-Lamia et al. 2012)
ΔΔ3	*ΔcopMRS::SpΩ ΔpcopMRS::C.K1*	*slr6039*, *slr6040*, *slr6041*, *sll0788*, *sll0789*, *sll0790*	This study
ΔΔ3RS	*ΔcopMRS::SpΩ ΔpcopR::C.K1 ΔglnN::P*_*copM*_*::copRS::Nat*	*slr6039*, *slr6040*, *slr6041*, *sll0788*, *sll0789*, *sll0790*, *slr0288*	This study
WTM	*ΔglnN::[C.K1::P*_*glnA*_*::copM]*	*slr0288*	This study
COPRM	*ΔcopMRS::SpΩ pcopR::C.C1 glnN::[C.K1::P*_*glnA*_*::copM]*	*slr0288*, *slr6039*, *slr6040*, *slr6041*, *sll0789*	This study
COPB	*copB::C.K1*	*slr6042*	(Giner-Lamia et al. 2012)

*Escherichia coli* DH5*α* cells were grown in Luria broth medium and supplemented with 100 *μ*g mL^−1^ ampicillin, 50 *μ*g mL^−1^ kanamycin, 20 *μ*g mL^−1^ chloramphenicol, and 100 *μ*g mL^−1^ spectinomycin when required.

### Insertional mutagenesis of *Synechocystis* genes

To generate the ΔΔ3 strain *copM* promoter was fused to the *copR* gene by overlapping polymerase chain reaction (PCR) using oligonucleotides Gcop-1/Gcop-2 and Gcop-3/Gcop-4, and cloned into pBS-SK+ to generate pCOPR67. A 2772-bp fragment *Nru*I-*Hind*III fragment was subcloned from cosmid cs1368 (Kazusa Research Institute) into pCOPR67 to generate pCOPRS9 (this plasmid contains *copM* promoter fused to *copRS* including the 780 bp downstream of *copS*). Then, a *Sac*I-*BstE*II fragment was excised from pCOPRS9, the plasmid was made blunt ended with the Klenow fragment and the SpΩ cassette was ligated to it generating pCOPRS11. This plasmid was used to transform the PCOP (Giner-Lamia et al. 2012) strain to generate the ΔΔ3 strain. To generate the ΔΔ3RS strain, a *Sal*I-*Hind*III fragment from pCOPRS9 was inserted in pGLNNpoly (which contains a synthetic *glnN* gene containing a synthetic polylinker; López-Maury L., Roldan M. and Florencio F.J.) generating pCOPRS10. A Nat resistance cassette (conferring nourseothricin resistance; Lopez-Maury et al. unpubl. ms.) was inserted into the *Hind*III site generating the pCOPRS10+. This plasmid was used to transform the ΔΔ3 strain to generate the ΔΔ3RS strain. For generation of the mutant strains, WTM and COPRM that express CopM constitutively, a 2174-bp fragment of the *glnN* gene was amplified from total genomic DNA using oligonucleotides glnNF and glnNR and was cloned into pGEMT to generate pGLNN. Then, a C.K.1 cassette (Cai and Wolk 1990) was inserted into *Eco*RV site of pGLNN generating pGLNN+. The glutamine synthetase *glnA* promoter (Reyes et al. 1997) and the whole *copM* sequence were fused by two-step PCR using oligonucleotide pGSF-pGSR and pCopMF-pCopMR, and cloned into the pGEMT, generating pQCOPM. Finally, a 669-bp *Knp*I-*Kpn*I fragment from pQCOPM was cloned into pGLNN+, generating pCOPMR+, which was used to transform WT and COPR strains to generate the WTM and the COPRM mutant strains, respectively.

### Cloning, purification, and metal-binding assays of CopM_(25-196)_ protein

A 519-bp band coding for the CopM_(25-196)_ (without the signal peptide) was PCR amplified from genomic DNA with oligonucleotides ACOPMSTF-COPMSTR, digested with *Kpn*I and *Sac*I, and cloned into pET51 digested with the same enzymes. CopM was expressed in *E. coli* BL21. A 1.5 L Luria broth culture was grown until the optical density at 600nm reached 0.6 when it was induced, with 0.2 mmol/L IPTG and incubated for 6 h at 25°C, cells were harvested by centrifugation and frozen at –20°C. Frozen pellets were resuspended in 40 mL of 100 mmol/L Tris HCl (pH 8), 150 mmol/L NaCl, 1 mmol/L BCSA, 1 mmol/L EDTA, and 2 mmol/L TCEP (buffer S) and broken by sonication. The suspension was centrifuged at 30,000*g* for 30 min at 4°C and the supernatant was loaded into a 5 mL streptavidin beads (IBA GmbH, Goettingen, Germany) column equilibrated in buffer S. Beads were washed with 50 mL of buffer S and CopM_(25-196)_ was eluted with 10 mL of 1× Strep-Tag elution buffer (IBA GmbH). CopM_(25-196)_ was further purified by gel filtration in a Hi-Load Superdex 75 (GE Healthcare, Freiburg, Germany) column equilibrated with 20 mmol/L Tris-HCl (pH 8) and 150 mmol/L NaCl. The purified protein was concentrated using a 3K Vivaspin concentrator (Merk Millipore, Darmstadt, Germany). Interaction of CopM_(25-196)_ with Cu, Ni, Zn, and Co was determined by immobilized metal ion affinity chromatography (IMAC). A 100-*μ*L aliquot of His-Bind resin (Novagen, Merk Millipore, Darmstadt, Germany) was loaded with 0.5 mL of 5 mmol/L of CuSO_4_, NiSO_4_, ZnSO_4_, or CoCl_2_ in water and then equilibrated in 25 mmol/L Tris-HCl (pH 8), 500 mmol/L NaCl (buffer A). A 100 *μ*g of purified CopM_(25-196)_ was applied to the columns. Unbound proteins were removed by washing with 2 mL of buffer A. Bound proteins were eluted with 100 *μ*L of 0.4 mol/L imidazole in buffer A. A quantity of 15 *μ*L of the imidazole eluted and flow-through fractions were analyzed by SDS-PAGE (sodium dodecyl sulfate polyacrylamide gel electrophoresis) and Coomassie brilliant blue (CBB) staining. Quantities of bound and unbound proteins were determined by densitometry.

The Cu(I) versus Cu(II)-binding preference in solution was determined following a protocol adapted from Burkhead et al. (2009). CopM_(25-196)_ (800 *μ*g) was incubated for 10 min at room temperature in buffer C (10 mmol/L Tris-HCl pH 7.5, 100 mmol/L NaCl) containing 0 or 1.5 mmol/L of CuSO_4_ in a volume of 500 *μ*L; for Cu(I)-binding experiments, ascorbic acid was added to 150 mmol/L final concentration to buffer C, and to the copper stock solution to reduce Cu(II) to Cu(I) ions. The mixture of protein and copper was incubated for 30 min at room temperature and loaded into a PD10 Desalting column (GE Healthcare), previously equilibrated with buffer C. Ten microliters was used before loading to the columns for protein determination by Bradford assay. Trichloracetic acid (100% w/v) was added to a final concentration of 10%, and the reaction was placed on ice for 10 min. The tubes were then centrifuged at 14,000*g* for 10 min at 4°C to separate the denatured protein. The supernatant, containing released copper ions, was then neutralized with 80 *μ*L of 6 mol/L NaOH and 100 *μ*L of 1 mol/L Tris buffer. After this, ascorbic acid was added again to 150 mmol/L final concentration, in this case to reduce all copper to Cu(I) ions. BCSA (Sigma-Aldrich Chemie, Steinheim, Germany), a chromophoric Cu(I) chelator, was added to a final concentration of 0.65 mmol/L to determine [Cu(BCS)_2_]^3−^ complex concentration using the previously reported extinction coefficient of 12,500 (mol/L)^−1^ cm^−1^ at 483 nm (Badarau and Dennison 2011), with a standard curve from 0 to 200 *μ*mol/L Cu(I). Either Cu(II)/Cu(I) or CopM incubated alone as a negative control and Bovine Serum Albumin (BSA) as positive copper-binding control were performed simultaneously under the same conditions. A second Cu(I)-binding test was performed with the addition of 1 mmol/L EDTA to remove any Cu(II) ions that might be present.

The apparent dissociation constant (*K*_D_) of Cu^I^-CopM_(25-196)_ was estimated via competition experiments as described previously using the chromophoric Cu(I) chelator bicinchoninic acid (BCA) (Djoko et al. 2007). BCA reacts with Cu(I) to form a stable 1:2 complex [Cu^I^(Bca)_2_]^3−^ with *β*_*2*_ = 10^17.2^ (mol/L)^−2^ at pH ≥7.0 (Djoko et al. 2007). The complex was quantified spectrophotometrically by absorbance at 358 nm using the previously reported extinction coefficient *ε*_358_ = 42,900 (mol/L)^−1^ cm^−1^ (Djoko et al. 2007). BCA (60 *μ*mol/L) in 20 mmol/L Tris-HCl (pH 7.5), 50 mmol/L NaCl (buffer B) was titrated against CopM_(25-196)_ (0–30 *μ*mol/L) in presence of 6 *μ*mol/L CopM. The samples were equilibrated for 5 min at room temperature before the measure in quartz cuvettes (Hellma, Merk Millipore, Darmstadt, Germany).

### Antibody production and western blotting

For CopM antibodies production, a 660-bp band coding for the complete sequence of CopM was PCR amplified from genomic DNA with oligonucleotides COPM1F-COPM1R, digested with *Nco*I and *Xho*I, and cloned into pET28 digested with the same enzymes. CopM was expressed in *E. coli* BL21. A 1.5 L Luria broth culture was grown until the optical density at 600nm reached 0.6 when it was induced with 0.2 mmol/L IPTG and incubated for 6 h at 30°C, cells were harvested by centrifugation and frozen at –20°C. Frozen pellets were resuspended in 40 mL of 25 mmol/L Tris-HCl (pH 8), 500 mmol/L NaCl (buffer A), and broken by sonication. The suspension was centrifuged 30 min at 30,000*g* at 4°C and the supernatant was loaded into a 1 mL His-Bind resin (Novagen) column loaded previously with 0.5 mL of 5 mmol/L with CuSO_4_ and equilibrated in buffer A. Columns were washed with 20 mL of buffer A and CopM was eluted with 1 mL of 0.4 mmol/L imidazole in buffer A. CopM was further purified by gel filtration in a Hi-Load Superdex 75 (GE Healthcare) column equilibrated with 20 mmol/L Tris-HCl (pH 8) and 150 mmol/L NaCl. The purified protein was concentrated using a 3K Vivaspin concentrator. Anti-CopM antisera was obtained according to the standard immunization protocols by injecting purified CopM protein in rabbits.

For western blot analysis, proteins were fractionated on SDS-PAGE and immunoblotted (Sambrook et al. 1989) with antibodies against: CopM (1:3000; this work), plastocyanin (1:12,000; Duran et al. 2004), thioredoxin A (1:3000; Navarro et al. 2000), or *Synechococcus* sp. PCC 6301 glutamine synthetase I (1:20,000; Merida et al. 1990). The ECL Plus immunoblotting system (GE Heathcare, Little Chalfont, UK) was used to detect the different antigens with anti-rabbit secondary antibodies conjugated to horseradish peroxidase (1:10,000). Films were scanned and quantified using ImageJ software.

### Periplasmic and extracellular fractions

Periplasmic fractions were prepared from *Synechocystis* as described previously (Fulda et al. 2000). Extracellular fractions were prepared from 20-mL samples of *Synechocystis* cultures in the mid-exponential growth phase (3–4 *μ*g chlorophyll mL^−1^). Extractions were performed by centrifugation of 20 mL of culture for 10 min at 4000*g* at 4°C. Supernatant was centrifuged (as above) twice and passed through a 0.2 *μ*L filter. Finally, the filtered extracellular fraction was concentrated using a 3K Vivaspin concentrator.

### Trypsin digestion and mass spectrometry analysis

CBB-stained proteins were excised from the gels, destained, dried, and rehydrated in 100 *μ*L 50 mmol/L NH_4_HCO_3_. A total of 15 *μ*L of 0.1 mg/mL trypsin in 1 mmol/L HCl was added and digestion was performed overnight at 37°C. Peptides were extracted with 20 *μ*L 0.5% Trifluoroacetic acid (TFA) and 0.5 *μ*L of each sample was applied onto the MALDI plate. Matrix-assisted laser desorption/ionization time of fligth mass spectrometry (MALDI-TOF MS) spectra were acquired on an Autoflex apparatus (Bruker Daltonics, Billerica, MA USA). External calibration was performed using Peptide Calibration Standard (Bruker Daltonics) and the trypsin autodigestion products of *m*/*z* values 842.5094 and 2211.1046 were used for internal calibration. Proteins were identified as the highest ranked result by searching the databases NCBInr or MSDB, including all species, using the MASCOT search engine (Matrix Science, London, UK). The mass tolerance was of 100 ppm and one missed cleavage was allowed. Carbamidomethylation of cysteines, oxidation of methionine, and acrylamide-modified cysteines were considered for PMF searches. For accepting the identification, the cutoff value for the Probability-Based Mowse score calculated by MASCOT (at *P* < 0.05) was used. For MS/MS data, the peptide mass tolerance was of 0.5 Da, MS/MS ion mass tolerance of 0.5 Da, allowance of one missed cleavage, and charge state +1.

### RNA isolation and northern blot analysis

Total RNA was isolated from 30-mL samples of *Synechocystis* cultures in the mid-exponential growth phase (3–4 *μ*g chlorophyll mL^−1^). Extractions were performed by vortexing cells in presence of phenol–chloroform and acid-washed-baked glass beads (0.25–0.3 mm diameter) as described previously (Garcia-Dominguez and Florencio 1997). Total RNA of 5 *μ*g was loaded per lane and electrophoresed in 1.2% agarose denaturing formaldehyde gels (Sambrook et al. 1989) and transferred to nylon membranes (Hybond N-Plus; GE Healthcare). Prehybridization, hybridization, and washes were in accordance with GE Healthcare instruction manuals. Probes for Northern blot hybridization were synthesized by PCR using oligonucleotide pairs: petEF-petER, copRF-copRR, copM1F-copM1R, copBF-copBR, all7633F-all7633R, all4988F-all4988R, all7594F-all7594R (see Table S2) for *petE*, *copR*, *copM*, *copB*, *all7633*, *all4988*, and *all7594*, respectively. As a control, in all cases, the filters were stripped and reprobed with a 580-bp *Hind*III-*Bam*HI probe from plasmid pAV1100 containing the constitutively expressed RNase P RNA gene (*rnpB*) from *Synechocystis* (Vioque 1992) or an *Anabaena* sp. PCC 7120 *rnpB* probe synthesized with oligonucleotides Ana_rnpBF and Ana_rnpBR. DNA probes were ^32^P labeled with a random-primer kit (GE Heathcare, Little Chalfont, UK) using [*α*-^32^P] dCTP (3000 Ci/mmol). Hybridization signals were quantified with a Cyclone Phosphor System (PerkinElmer, Waltham, Massachusetts, USA). Each experiment was performed at least two independent times.

### Determination of intracellular copper content

For intracellular copper content, 600 mL of exponentially growing cells were treated with 1 *μ*mol/L of copper for 5 h. Cells were centrifuged at 5000*g*, washed twice with BG11C-Cu, and dried overnight in an oven at 85°C. Hundred milligrams of dried cells was microwave digested, dissolved in suprapure HNO_3_, and analyzed by ICP in an ICP-OES Varian ICP 720-ES. Copper content was normalized to phosphorus contents and compared to WT copper accumulation. Data shown represent the average ± standard error from three biological independent experiments.

## Results

### *CopM gene* codes for a copper-induced periplasmic protein

**Figure 1:**
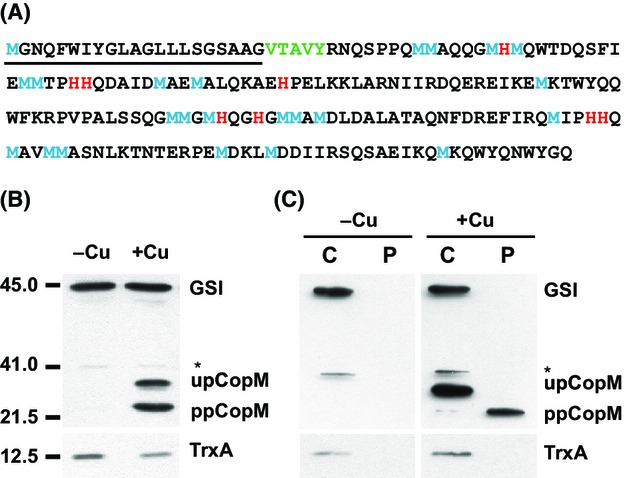
CopM is a periplasmic protein induced by copper. (A) CopM amino acid sequence. The signal peptide sequence is underlined, the most likely cleavage site is shown in green, methionine and histidine residues are shown in blue and red, respectively. (B) Western blot analysis of CopM in the presence or absence of copper. WT cells were grown in BG11C-Cu medium to mid-log growth phase and exposed for 4 h to copper 1 *μ*mol/L. Five micrograms of total protein from soluble extracts was separated by 15% SDS-PAGE and analyzed by western blot to detect CopM, thioredoxin A (TrxA), and glutamine synthetase type I (GSI). (C) Western blot analysis of CopM cellular localization. WT cells were grown in BG11C-Cu medium to mid-log growth phase and exposed for 4 h to copper 1 *μ*mol/L. Five micrograms of both cytosolic (C) and periplasmic (P) protein from soluble extracts was separated by 15% SDS-PAGE and analyzed by western blot to detect CopM, TrxA, and GSI. upCopM, unprocessed protein; ppCopM, processed protein.

*CopM* (*sll0788*) and *pcopM* (*slr6038*) gene products are annotated as hypothetical 21.5 kDa proteins (196 aa and 99% identity) that contain two DUF305 domains of unknown function. The sequence analysis of CopM showed a hydrophobic sequence of 23 aa in its N-terminal region, predicted as a signal peptide (0.976 probability according to SignalP 3.0 Server), with the most likely cleavage site 24–25 position (VTA-VY; Fig.1A), suggesting that *copM* codes for a periplasmic protein. In order to demonstrate this, we generated specific antibodies against the complete CopM sequence and used them to analyze its expression in response to 1 *μ*mol/L copper, a noninhibitory concentration that showed a strong transcriptional response of the *copMRS* operon (Giner-Lamia et al. 2012, 2014). Three different bands were detected in total cells from copper-treated cultures but only two of them were copper inducible (Fig.1B). The two induced bands corresponded to the predicted size of the full length (upCopM, 22.5 kDa) and the processed form (ppCopM, 20.2 kDa) of CopM, respectively, while the third one corresponded to a cross-reacting protein recognized by the anti-CopM sera (Fig.1B and C). In contrast, when cytoplasmic and periplasmic protein fractions were analyzed by western blot, upCopM was only detected in the cytoplasmic fraction and the faster migrating band (corresponding to ppCopM) was detected almost exclusively in the periplasmic fraction (Fig.1C). As a control for cytoplasmic contamination of the periplasmic fraction preparation, we tested for the presence of two abundant cytosolic proteins, glutamine synthetase (Merida et al. 1990), and thioredoxin A (Navarro and Florencio 1996; Florencio et al. 2006). These two proteins were only detected in the cytosolic fraction (Fig.1C). Additionally, and to further confirm the purity of the periplasmic fractions, three different bands from a CBB-stained gel were identified by MALDI-TOF, after in gel trypsin digestion of the excised bands (Fig. S1). These three bands were identified using MASCOT as three previously identified periplasmic proteins: a C-terminal peptidase (*slr1751*; Fulda et al. 2000), an iron-binding protein FutA2 (*slr0513*; Waldron et al. 2007), and a *β*-type carbonic anhydrase (*slr0051*; Fulda et al. 2000; Fig. S1 and Table S1). These results suggest that both *copM* (*sll0788*) and *pcopM* (*slr6038*) genes code for a periplasmic protein.

### Copper-dependent regulation of CopM

**Figure 2:**
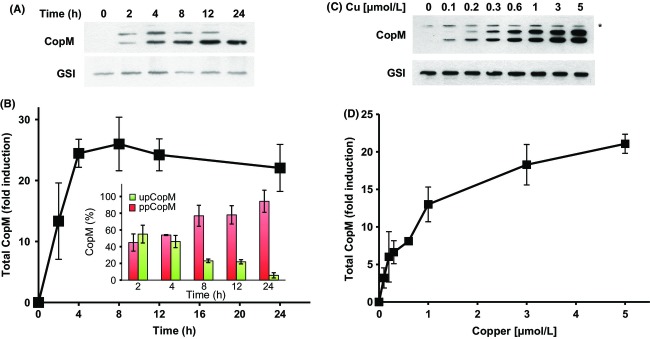
CopM accumulates in response to copper in a time- and dose-dependent manner. (A) Western blot analysis of CopM levels for 24 h after copper addition in the WT strain. Cells were grown in BG11C-Cu medium to mid-log growth phase and exposed for 24 h to copper 1 *μ*mol/L. Five micrograms of total protein from soluble extracts was separated by 15% SDS-PAGE and analyzed by western blot to detect CopM, and glutamine synthetase type I (GSI). (B) Quantification of total CopM levels in response to copper addition in the WT strain. Western blot signal of three independent experiments as the one shown in A were quantified using ImageJ program. CopM levels were normalized to the GSI signal. Error bars represent SE. *Inset*: Quantification of the relative proportion of ppCopM (red bars) and upCopM (green bars). (C) Western blot analysis of CopM levels after the addition of different copper concentrations in the WT strain. Cells were grown in BG11C-Cu medium to mid-log growth phase and exposed for 3 h to the indicated copper concentration. Five micrograms of total protein from soluble extracts was separated by 15% SDS-PAGE and subjected to western blot to detect CopM and GSI. (D) Quantification of total CopM levels in response to different copper concentrations in the WT strain. Western blot signal of three independent experiments as the one shown in C was quantified using ImageJ program. CopM levels were normalized to the GSI signal. Error bars represent SE.

In our previous study, we determined that *copMRS* was highly expressed in response to copper but not to other metals (Giner-Lamia et al. 2012). According to this, we have analyzed *copM* expression in response to 1 *μ*mol/L of copper for 24 h and to different copper concentrations in the WT strain (unfortunately we cannot distinguish between the two copies of these genes because of their high level of identity (>93% at nucleotide level) and we will refer to them simply as *copM* when analyzing gene expression, although both copies have shown to be transcribed (Nagarajan et al. 2012; Giner-Lamia et al. 2014). As is shown in Figure2A and B, total CopM protein was highly induced after the addition of 1 *μ*mol/L of copper, with a maximum peak of accumulation around 4–12 h (with a maximum induction of 26.15 ± 4.2-fold) that corresponded mainly to ppCopM. The increase in ppCopM levels with respect to upCopM appears after 4 h and at 24 h ppCopM accounted for 98% of the total CopM protein in the cell, indicating that the processed protein was the main accumulated form in response to copper. In the case of *copM* transcript, it followed a similar accumulation kinetics during the first 8 h (Fig. S2A and S2B) but decreasing afterward. The fact that the level of total CopM was practically unaffected after 12 h despite reduced levels of *copM* transcript, suggests that the protein could be stabilized in the presence of copper. This result is in agreement with a possible role of CopM as a metallochaperone and/or as a copper buffer system, which will bind and block free copper into the periplasm. To gain a better insight into CopM regulation, we have also analyzed the impact of copper concentration in CopM expression. Exponentially growing WT *Synechocystis* cells were challenged with different copper concentrations, from 0.1 to 5 *μ*mol/L of copper for 3 h, a time in which transcript levels were still high (Fig.2C and D). In this case, accumulation of both protein and RNA was very similar reaching the maximum fold induction at 5 *μ*mol/L of copper, showing a direct correlation between the amount of transcript and protein (Figs.2C and D, S2C and S2D). Notably, at the lower copper concentration used, 0.1 *μ*mol/L, we only observed the ppCopM form and almost the same *copM* transcripts levels than in the absence of copper, suggesting that after 3 h this copper concentration could be completely managed by ppCopM in the periplasm avoiding further activation of the CopRS two-component system. Finally, we have also analyzed the accumulation of *copB* transcript that showed lower inductions than *copM* in all copper concentrations tested (Fig. S2C and S2D), suggesting that at low copper concentration, CopM is the main component of the *cop* system acting against copper because *copBAC* is almost not expressed. These data point out that CopM and CopBAC are two systems that work independently managing the copper in the cell.

### CopM is implicated in copper resistance

**Figure 3:**
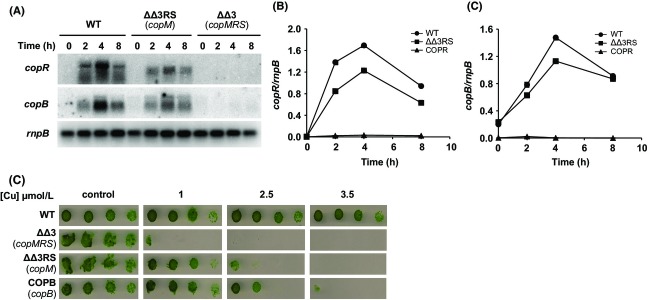
A mutant strain lacking CopM is sensitive to copper. (A) Northern blot analysis of *copR* and *copB* expression in response to copper addition in the WT, ΔΔ3RS, and ΔΔ3 strains. Total RNA was isolated from WT, ΔΔ3RS, and ΔΔ3 strains grown in BG11C-Cu medium to mid-log growth phase and exposed for 8 h to copper 1 *μ*mol/L. Samples were taken at the indicated times. The filter was subsequently hybridized with *copB*, *copR*, and *rnpB* (as a loading control) probes. (B) Quantification of the relative mRNA levels of *copM* in response to copper addition in the WT, ΔΔ3RS, and ΔΔ3 strains. Radioactive signals of two independent experiments (as it is shown in A) for each strain were quantified and averaged. RNA levels were normalized with the *rnpB* signal. Plots of relative mRNA levels versus time were drawn. (C) Quantification of the relative mRNA levels of *copB* in response to copper addition in the WT, ΔΔ3RS, and ΔΔ3 strains. Radioactive signals of two independent experiments (as it is shown in A) for each strain were quantified and averaged. RNA levels were normalized with the *rnpB* signal. Plots of relative mRNA levels versus time were drawn. (D) Phenotypic characterization of mutant strains affected in *cop* genes. Tolerance of WT, ΔΔ3RS, ΔΔ3, and COPB strains to copper was examined. Ten-fold serial dilutions of 1 *μ*g chlorophyll mL^−1^ mid-log phase cells suspension were spotted onto BG11C-Cu supplemented with the indicated copper concentrations. Plates were photographed after 5 days of growth.

**Figure 4:**
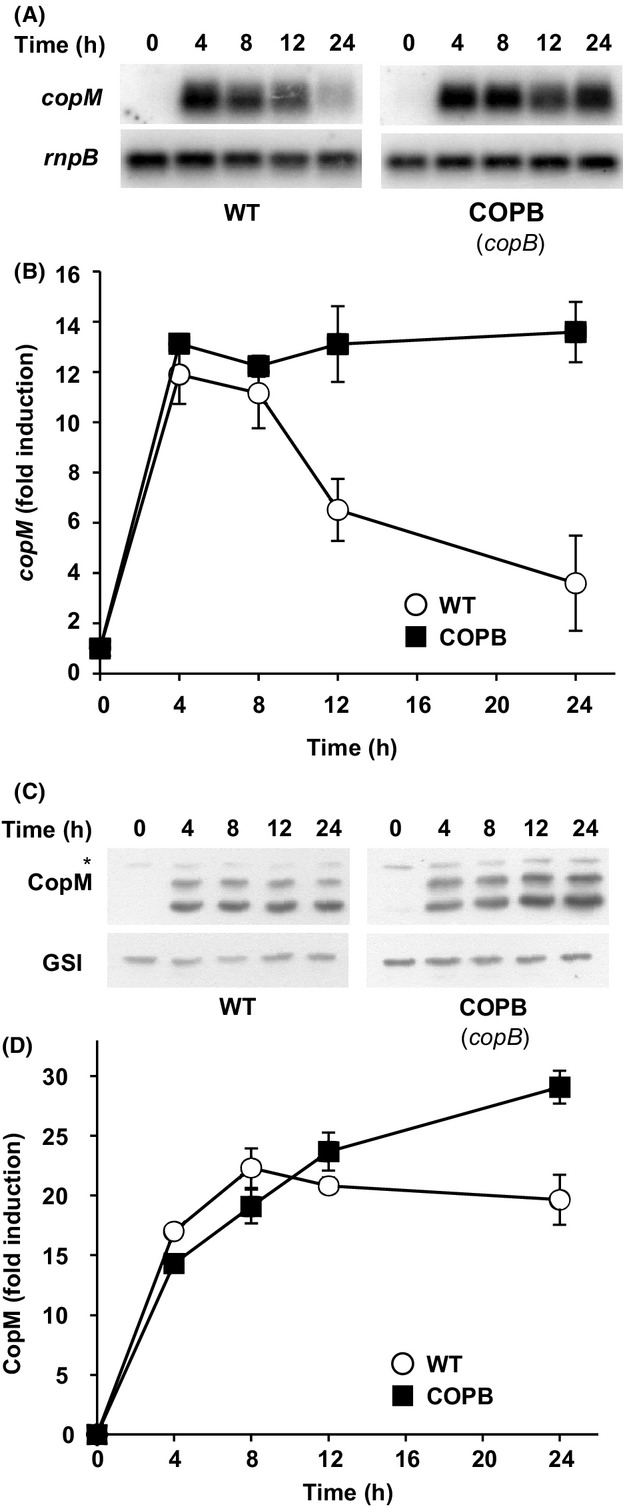
Absence of CopBAC efflux system leads to a higher accumulation of *copM* transcript and CopM protein. (A) Northern blot analysis of *copM* expression in response to copper addition in WT and COPB strains. Total RNA was isolated from WT and COPB cells grown in BG11C-Cu medium to mid-log growth phase and exposed for 24 h to copper 1 *μ*mol/L. Samples were taken at the indicated times. The filter was hybridized with *copM* probe and subsequently stripped and rehybridized with an *rnpB* probe as a control. (B) Quantification of the relative mRNA levels of *copM* in response to copper addition in the WT (white circles) and the COPB (black squares) strains. Radioactive signals of three independent experiments for each strain were quantified and averaged. RNA levels were normalized with the *rnpB* signal. Plots of relative mRNA levels versus time were drawn; error bars represent SE. (C) Western blot analysis of CopM levels after copper addition in WT and COPB strains. Cells were grown in BG11C-Cu medium to mid-log growth phase and exposed for 24 h to copper 1 *μ*mol/L. Five micrograms of total protein from soluble extracts was separated by 15% SDS-PAGE and analyzed by western blot to detect CopM and GSI. (D) Quantification of CopM levels in response to copper addition in WT (white circles) and COPB (black squares) strains. Western blot signal of three independent experiments were quantified using ImageJ program. CopM levels were normalized with the GSI signal. Error bars represent SE.

In order to investigate the role of CopM in copper resistance in *Synechocystis*, a mutant lacking *copM* was constructed in two steps. First of all, we generated a strain lacking completely the two copies of *copMRS* operon by deleting both the chromosome and the plasmid copies of these genes, that we named ΔΔ3 strain (Fig. S3A). Second, we generated a plasmid lacking *copM* in which *copRS* were under the control of *copMRS* promoter region but in which *copM* was not present. This construct was inserted in the *glnN* locus (a nonessential gene in the conditions used here; Muro-Pastor et al. 2001; Reyes and Florencio 1994; Sauer et al. 2000) of the ΔΔ3 strain generating the ΔΔ3RS strain (Fig. S3B). Surprisingly, the complete segregation of this strain was unsuccessful since all the colonies obtained were merodiploids as we always detected the band corresponding to the WT *glnN* gene without the insert (Fig. S3C). To test whether the lack of *copM* affected *copRS* function in the ΔΔ3RS mutant strain, the induction of both *copR* and *copB* in response to copper was analyzed by northern blot experiments. As is shown in Figure3A, the *copRS* transcript size in ΔΔ3RS strain was smaller when compared to the WT strain *copMRS* transcript, due to the lack of *copM* gene. Despite this, the induction kinetics of *copRS* in the ΔΔ3RS strain was quite similar to that of *copMRS* in the WT strain, although the levels were slightly reduced (Fig.3A and B). A similar effect was also observed in the case of *copBAC* operon, the induction was slightly lower at 4 h in the ΔΔ3RS strain compared to the WT strain, but the kinetics were almost identical (Fig.3A and B). Finally, as expected, the ΔΔ3 strain did not show transcripts for any of these genes (Fig.3A–C). Once we have established that *copRS* were expressed in the *copM* mutant, we wanted to examine whether the absence of *copM* had an effect on copper resistance. For this purpose, cells of WT, ΔΔ3, ΔΔ3RS, and COPB (a mutant strain affected in the first gene of the *copBAC* operon) strains were cultured to mid-log phase and spotted into plates containing different copper concentrations. The ΔΔ3RS and COPB strains showed a similar sensitivity to copper, with reduced growth at 2.5 *μ*mol/L of copper (Fig.3B), while the ΔΔ3 failed to grow at 1 *μ*mol/L, indicating that both the CopM protein and the CopBAC efflux system, contribute to copper resistance in *Synechocystis*. The fact that the copper sensitivity of the ΔΔ3 strain (and the COPR strain; see below and Giner-Lamia et al. 2012), which do not express neither *copM* nor *copBAC*, was higher than the sensitivity of mutants lacking only CopM (ΔΔ3RS strain) or CopBAC (COPB strain), reinforces the idea that these two elements could work as two independent systems. To gain a better insight into this, we analyzed whether the absence of CopBAC efflux system (using the COPB strain) had any effect on the expression levels or the induction kinetics of *copM*. For this, we analyzed the induction of *copM* transcript and CopM protein accumulation for 24 h after 1 *μ*mol/L copper addition in both the WT and the COPB strains. Northern and western blot analysis showed that *copM* transcript accumulation correlated with CopM accumulation in both strains (Fig.4). The WT strain showed a decline in *copM* transcript (after 12 h) and accumulation of CopM remained constant afterwards (Fig.4A and B). By contrast, the COPB strain maintained a higher transcript level of *copM* after 8 h, which leads to a higher accumulation of CopM protein (about 20% more) after 12 h compared to the WT strain (Fig.4C and D). These results show that the absence of CopBAC induces *copM* expression at higher levels in the long term and this could explain its higher resistance when compared with COPR or ΔΔ3 strains.

### Constitutive expression of CopM partially restores copper resistance in the COPR mutant strain

**Figure 5:**
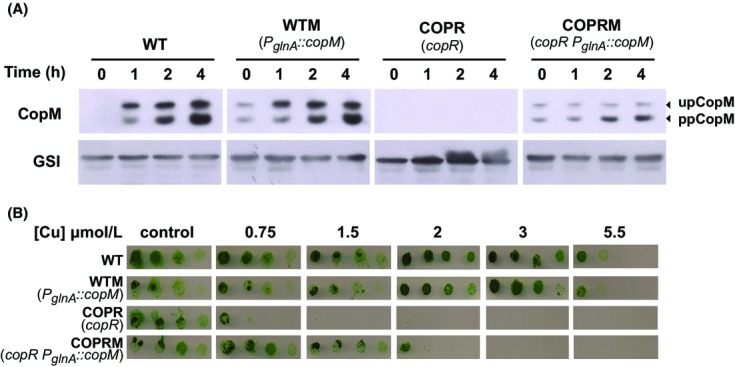
Constitutive expression of CopM partially restores copper resistance of COPR mutant strain. (A) Western blot analysis of CopM levels after copper addition in WT, WTM, COPR, and COPRM strains. Cells were grown in BG11C-Cu medium to mid-log growth phase and exposed for 4 h to 1 *μ*mol/L copper. Five micrograms of total protein from soluble extracts was separated by 15% SDS-PAGE and analyzed by western blot to detect CopM and GSI. (B) Phenotypic characterization of mutant strains affected in *cop* genes. Tolerance of WT, WTM, COPR, and COPRM strains to copper was examined. Ten-fold serials dilutions of a suspension of 1 *μ*g chlorophyll mL^−1^ cells grown to mid-log phase were spotted onto BG11C-Cu supplemented with the indicated copper concentrations. Plates were photographed after 5 days of growth.

Owing that all our attempts to get fully segregated mutant strains lacking all copies of *copM* failed and to further investigate the function of CopM in copper homeostasis, we decided to carry out a different approach generating a mutant strain that expressed constitutively the CopM protein (Fig. S4). For that, a copy of the *copM* under the control of the *glnA* promoter (which is expressed constitutively in media containing NO_3_) was introduced in the *glnN* locus of both the WT and the COPR strains generating the WTM and COPRM strains, respectively. Complete segregation of these mutants was verified by PCR (Fig. S4B). To confirm that CopM was expressed constitutively in these two mutant strains, western blot analysis was performed from total cells of WT, WTM, COPR, and COPRM cultured in BG11C-Cu and exposed for 4 h to 1 *μ*mol/L of copper. In both WTM and COPRM strains, CopM was detected before copper addition, while in the WT strain the protein was only observed after copper addition (Fig.5A). As we expected, the protein was not detected in the COPR strain. In both WT and WTM strains both CopM forms levels increased after copper addition, in agreement with induction of *copM* transcript levels, although the processed form was accumulated at higher levels (Fig.5A). Remarkably, the COPRM strain showed a clear increase in the ppCopM after copper addition while the upCopM levels remained constant. These results suggest that the processed form of the protein was stabilized after copper addition. In order to test whether CopM expression could restore copper resistance in mutants lacking the CopRS system or increase WT copper tolerance the growth of WT, WTM, COPR, and COPRM strains was analyzed in the presence of different copper concentrations in solid media. While the COPR strain growth was affected at 0.75 *μ*mol/L of Cu, as previously reported (Giner-Lamia et al. 2012), the COPRM strain was able to grow up to 1.5 *μ*mol/L (Fig.5B), suggesting that expression of CopM in COPRM strain was able to partially restore COPR copper tolerance. In contrast, an increase in copper tolerance in the WTM strain compared to the WT strain was not observed, suggesting that *copM* expression from its endogenous promoter is enough to manage the copper concentrations tested in this experiment.

### CopM is a copper-binding protein

**Figure 6:**
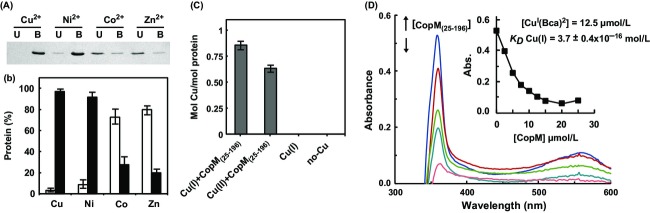
CopM binds copper in vitro. (A) Analysis of CopM_(25-196)_ protein interaction with metals. His-Bind resin columns were loaded with 5 mmol/L CuSO_4_, NiSO_4_, ZnSO_4_, and CoCl_2_. Hundred micrograms purified CopM_(25-196)_ protein was applied to the columns. The unbound (U lanes) and bound (B lanes) fractions were analyzed by 15% SDS-PAGE and Coomassie blue staining. (B) Quantification of CopM_(25-196)_ in bound and unbound fractions. Coomassie-stained gel was scanned and bands intensity was quantified using ImageJ program; the graph represents the average of three independent experiments. Unbound fraction (white), bound fraction (black). (C) Specificity of copper ion binding by CopM_(25-196)_. CopM of 800 *μ*g was incubated for 10 min with 1 mmol/L Cu(I) or Cu(II) and gel filtration was used to remove unbound Cu ions, which were measured by titration with BCS as described in “Experimental Procedures” section. Bar graph represents mol of Cu/mol of CopM from three independent experiments. Error bars represent SE. Cu(I), only copper; no-Cu only CopM_(25-196)_. (D) Determination of the Cu(I) dissociation constant, *K*_D_, of CopM_(25-196)_ by titration into a solution of 60 *μ*mol/L BCA. The graph shows the spectral changes of the [Cu(BCA)_2_]^3−^ form on the CopM_(25-196)_ titration. BCA (60 *μ*mol/L) in 20 mmol/L Tris-HCl (pH 7.5), 50 mmol/L NaCl was titrated against CopM (0–25 *μ*mol/L) measuring absorbance in the 350–600 nm range. *Inset*: The decrease at 358 nm relative to CopM_(25-196)_ additions for a [Cu(BCA)_2_]_FINAL_ = 1.4 *μ*mol/L. Dissociation constant, *K*_D_, was estimated as described in “Experimental Procedures” section from three independent experiments like the one shown in D.

As mentioned above, CopM contains two domains of unknown function DUF305, which are characterized by the presence of a conserved HH motif that is present in a small family of secreted proteins in bacteria (Finn et al. 2014). In addition, the CopM protein sequence contains a high number of histidine (8) and methionine (23) residues (Fig.1A), which are usually implicated in direct metal binding in proteins. To test whether CopM was able to bind metals, we analyzed the interactions of the recombinant protein (a strep tagged version lacking the transit peptide, CopM_(25-196)_) with metals ions using metal chromatography (Fig.6). High levels of CopM_(25-196)_ were retained by beads charged with 5 mmol/L of Cu(II) and Ni(II), while almost no protein was retained by Zn(II) and Co(II) charged beads (Fig.5A and B). To further investigate CopM interaction with copper, we analyzed its specificity for Cu(I) (reduced with ascorbate) versus Cu(II). For that, purified CopM_(25-196)_ protein was incubated with copper ions in solution and unbound copper ions were removed by gel filtration. In order to quantify the copper bound to CopM_(25-196)_, proteins in the fractions from the gel filtration were precipitated with TCA, the supernatant was reduced by ascorbate and the copper concentration was determined by BCA (a chromogenic copper chelator that can be quantified by absorbance at 358 nm; Djoko et al. 2007; Xiao and Wedd 2010). CopM_(25-196)_ was able to bind both copper ions in solution with an approximate 1:1 ratio (Fig.5C), although binding to Cu(I) was more stable than to Cu(II) ions. Incubation of either copper or the protein alone did not show any copper presence after gel filtration and copper determination with BCA (Fig.5C). This result is consistent with the high number of methionine residues that are present in the CopM sequence. Cu(I) has a high affinity for Cys and Met residues in proteins, and Cu(I) binding and transport proteins are usually enriched in these residues (Su et al. 2011).

In order to obtain a precise Cu(I) binding affinity of CopM, a series of titrations of Cu(I)-loaded BCA with increasing amounts of CopM_(25-196)_ were performed (Fig.5D). This revealed a CopM_(25-196)_ concentration-dependent decrease in BCA-Cu(I) concentration that allowed us to calculate an apparent dissociation constant (*K*_Dapp_) for CopM of 3.7 ± 0.4 × 10^−16^. All these data demonstrate that CopM is able to bind copper in vitro, and it shows high affinity toward Cu(I), probably mediated by the high number of methionine residues present in its sequence.

### CopM affects copper metabolism

**Figure 7:**
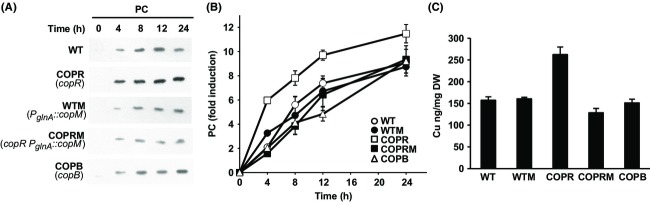
Constitutive expression of CopM restores both plastocyanin and cellular copper levels. (A) Western blot analysis of plastocyanin levels after copper addition in WT, WTM, COPR, COPRM, and COPB strains. Cells were grown in BG11C-Cu medium to mid-log growth phase and exposed for 24 h to copper 1 *μ*mol/L. Five micrograms of total protein from soluble extracts was separated by 15% SDS-PAGE and analyzed by western blot to detect plastocyanin. (B) Quantification of plastocyanin levels in response to copper addition in WT (white circles), WTM (black circles), COPR (white squares), COPRM (black squares), and COPB (white triangles) strains. Western blot signal of three independent experiments was quantified using ImageJ program. Plastocyanin levels were normalized to the GSI signal. Error bars represent SE. (C) Total intracellular copper contents in WT, WTM, COPR, COPRM, and COPB strains. Cells were grown in BG11C-Cu medium to mid-log growth phase and exposed for 1 *μ*mol/L of copper for 5 h. Cells were centrifuged, washed twice with BG11C-Cu, and dried. Hundred micrograms of dried cells was microwave digested, dissolved in suprapure HNO_3_, and analyzed by ICP. Error bars represent SE from three independent experiments.

Plastocyanin is the main copper containing protein in *Synechocystis* and is located in the thylakoid lumen (Tottey et al. 2001, 2002, 2012). Previous studies indicated that mutants affected in copper transport across the two membranes, lacking either PacS or CtaA, the two P_I_-type ATPases that deliver copper to plastocyanin, have reduced plastocyanin levels (Tottey et al. 2001, 2012) and that the double mutant does not express the *petE* gene in the presence of copper (Giner-Lamia et al. 2012). Having established that CopM was able to directly bind copper and was implicated in copper resistance, we wanted to investigate whether there was any interaction between plastocyanin levels and the presence of both CopM and CopBAC efflux systems. For that, we monitored plastocyanin levels in the WT, COPB, COPR, WTM, and COPRM mutant strains for 24 h after the addition of copper 1 *μ*mol/L to cells grown in the absence of copper (Fig.7A). Although *petE* transcript levels were the same in all strains (Fig. S5), the plastocyanin protein levels in the COPR strain was higher than in the other strains (Fig.7A and B), suggesting that in the absence of both *cop* systems more copper is available for plastocyanin synthesis. These data are in agreement with the fact that COPR cells accumulated about twice the amount of copper than WT cells (Giner-Lamia et al. 2012), and raises the possibility that plastocyanin could act as a sink for copper in *Synechocystis* cells. To further investigate the role of CopM in copper metabolism, we wanted to test whether constitutive expression of *copM,* in both WTM and COPRM strains, could affect plastocyanin accumulation. As shown in Figure7A and B, the constitutive expression of *copM* restored plastocyanin protein levels in the COPRM strain to WT levels. However, we did not detect any differences in the amount of plastocyanin in the case of the WTM strain. Neither was the plastocyanin levels affected in the COPB strain (Fig.7A), which compensated the absence of CopBAC system efflux by a higher induction of CopM protein in presence of copper (Fig.3). These results suggest that the presence of CopM could interfere with the copper supply to plastocyanin synthesis. To test this hypothesis, we analyzed the amount of intracellular copper accumulated in these strains after the addition of copper 1 *μ*mol/L (Fig.7C), expecting that strains which had increased CopM levels will show higher copper content. However, and contrary to what we expected, the total cellular copper content correlated only with plastocyanin levels, but not with CopM levels and therefore only the COPR strain showed higher copper content. This result suggests that CopM is able to reduce cellular copper levels but this is not a consequence of direct copper accumulation and immobilization in the periplasm, because in this case the COPRM strain would have similar copper content to the COPR strain. Furthermore, all these data combined with the fact that CopM increased copper tolerance in the COPRM strain, suggest that the role of CopM could be related to either avoiding copper import in the cell or assisting copper transport outside the cell by a mechanism independent of CopBAC, which is not expressed in the COPRM strain (Fig. S6).

### A fraction of CopM is localized to extracellular space

**Figure 8:**
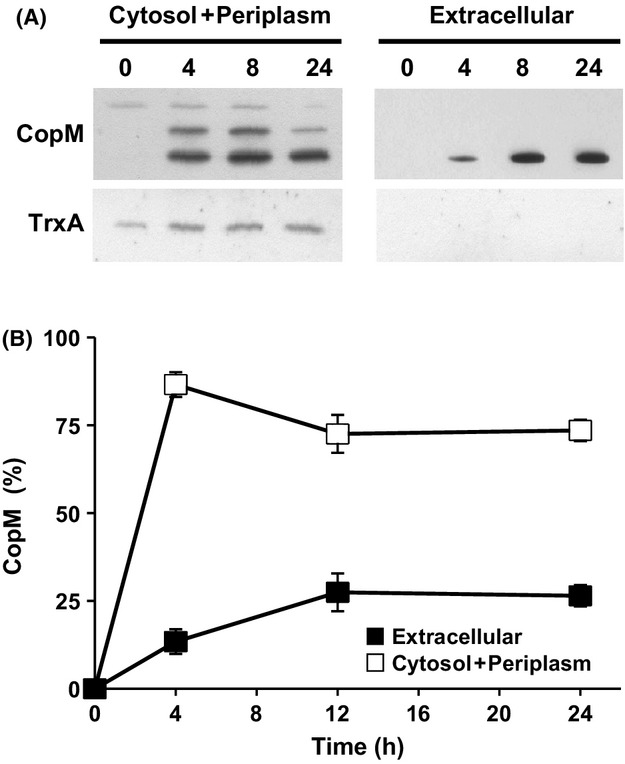
CopM is localized in both periplasmic and extracellular space. (A) Western blot analysis of CopM in cellular and extracellular fractions. WT cells were grown in BG11C-Cu medium to mid-log growth phase and exposed for 24 h to copper 1 *μ*mol/L. Five micrograms of cytosol + periplasm or extracellular protein from soluble extracts was separated by 15% SDS-PAGE and subjected to western blot to detect CopM and TrxA as loading control. (B) Quantification of CopM levels in response to copper in cellular and extracellular fractions. Western blot signal of three independent experiments were quantified using ImageJ program. Error bars represent SE.

All the aforementioned results suggested that CopM could be directly involved in avoiding copper uptake. Because the DUF305 domains are present in some bacterial secreted proteins, we wanted to test whether CopM was also present outside the cell. For that, WT *Synechocystis* cells were grown for 24 h in presence of copper 1 *μ*mol/L and both extracellular and total cellular protein fractions were analyzed. To avoid any cell (and cytoplasmic) contamination in the extracellular fraction, cells were gently centrifuged (4000*g*) and the media were filtered twice through a 0.2 *μ*m filter before concentration and western blot analysis. An abundant cytosolic protein of a similar size of CopM, TrxA, was used as a control of cellular contamination in the extracellular fraction. As shown in Figure8A, while upCopM and TrxA were only detected in the cellular fraction, the processed band of CopM was detected in both extracellular and cellular fractions. After 24 h ∼30% of total CopM protein (in the ppCopM form) appeared in the extracellular space (Fig.8B), indicating that an important fraction of the protein was localized outside the cell. These data suggest that the reduction in the intracellular copper in the COPRM strain (Fig.7C) could be mediated by either direct copper binding by CopM in the extracellular space and/or copper loading in the periplasm and its export outside the cell.

### Transcriptional analysis of *copM* homologs in *Anabaena* sp. PCC 7120

**Figure 9:**
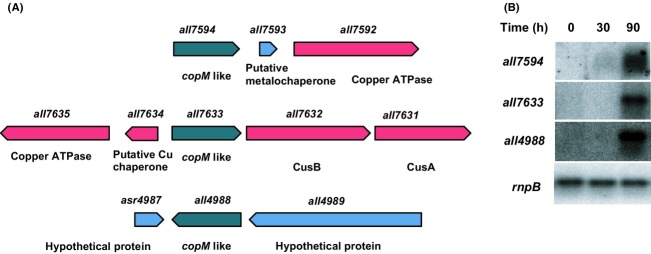
Transcriptional analysis of genes with homology to *copM* in *Anabaena* sp. PCC 7120. (A) Schematic representation of the genomic regions of *Anabaena* sp. PCC 7120 *copM* homologs. (B) Northern blot analysis of the expression of *all7594*, *all7633*, and *all4988* in response to copper addition in *Anabaena* sp. PCC 7120 WT strain. Total RNA was isolated from cells grown in BG11C-Cu medium to mid-log growth phase and exposed for 90 min to copper 1 *μ*mol/L. Samples were taken at the indicated times. The filter was subsequently hybridized with *all7594*, *all7633*, *all4988*, and *rnpB* (as a loading control) probes.

A BlastP search of CopM sequence returned several genes coding for CopM homologs in other cyanobacterial genomes. All of these homologous genes contain at least one DUF305 domains. In most cases, the CopM-like genes were located in the vicinity of other copper-related genes such us RND efflux system, putative copper ATPases, or putative copper chaperones suggesting that these genes could function in copper metabolism. In the case of *Anabaena* sp. PCC 7120, three CopM-like genes were found: *all7594*, *all7633*, and *all4988* which displayed an identity at the protein level of 50.1%, 43.8%, and 40.1%, respectively. All of these open reading frames (ORFs) contain two copies of DUF305 domains as CopM and with the exception *all4988*, they were close to putative copper-related genes. *all7594* was upstream to genes coding for a putative metallochaperone and a putative copper ATPase (Fig.9A). *all7633* was also located next to and in opposite orientation to a putative copper metallochaperone and copper ATPase-like transporter, and it was upstream to two genes of a RND transport system (*all7632* and *all7631*; Fig.9A), which were previously reported to be expressed in copper-containing media and repressed in the presence of iron (Nicolaisen et al. 2010). In order to analyze the transcriptional response to copper of these three genes, *Anabaena* sp. PCC 7120 cells were grown in BG11C-Cu, 3 *μ*mol/L copper was added, and expression of these three genes was analyzed by northern blot. As it can be observed in the Figure9B, *all7594*, *all7633*, and *all4988* were similarly induced after copper addition. All these data indicate that these genes could have a conserved role in copper homeostasis in *Anabaena* sp. PCC 7120.

## Discussion

This work shows that *copM* (which is part of the *copMRS* operon) codes for a protein involved in copper resistance in *Synechocystis*. Mutants completely lacking the *copM* gene (ΔΔ3RS strain) were more sensitive to copper in the media than WT cells (Fig.3), although they were more resistant to copper than mutants lacking the CopRS two-component system (ΔΔ3 or COPR strains; Fig.3) and showed similar resistance levels to mutants in the CopBAC transport system. Furthermore, constitutive expression of *copM* in a COPR strain (which lacks a functional CopRS two-component system and therefore does not express neither the *copBAC* operon nor the *copM* gene; Giner-Lamia et al. 2012) partially suppressed the copper sensitivity phenotype of this strain (Fig.5). In addition, while *copMRS* was induced even at nontoxic copper concentrations, *copBAC* was only expressed at higher concentrations of this metal (Fig. S2), suggesting that CopBAC is only needed when CopM is overloaded with copper. Finally, both *copM* transcript and CopM protein showed higher levels of expression in a mutant lacking *copB* (Fig.4; COPB strain), which suggest that the inability to detoxify copper, by the absence of a functional CopBAC system, causes a higher induction of *copM*. All together these data support an important role of CopM in copper resistance in *Synechocystis* and that it constitutes an independent copper resistance mechanism to the CopBAC efflux system. A similar situation was observed in *E. coli* where the *cus* system is expressed under conditions in which the *cue* system is overloaded or not functional (e.g., during anaerobiosis; Grass and Rensing 2001b; Franke et al. 2003; Rensing and Grass 2003).

Additional evidence for the role of CopM in copper homeostasis comes from our finding that CopM was exported to the periplasm (Fig.1) and was able to bind copper (Fig.6). Purified CopM showed a preference for Cu(I) over Cu(II), although in both cases it probably binds ∼1 Cu atoms per monomer. Unfortunately, we were unable to obtain an affinity constant for the Cu(II)-CopM interaction (because its affinity for Cu(II) was very low), but we were able to use Cu(I) binding affinity assays to calculate the Cu(I)-CopM *K*_D_ which was 3.7 ± 0.4 × 10^−16^ (Fig.6), indicating a high CopM-Cu(I) affinity. This is consistent with the high number of Met (23) and His (8) residues present in the CopM sequence and the absence of cysteines which are common features in proteins that bind Cu(I) in an oxidizing environment like the periplasm (Puig et al. 2002; Jiang et al. 2005; Davis and O'Halloran 2008). Binding of both copper forms has been reported for other metallochaperones that are involved in copper detoxification such as CopC, PcoC, or CopK (Arnesano et al. 2003; Wernimont et al. 2003; Djoko et al. 2007; Bersch et al. 2008; Chong et al. 2009; Sarret et al. 2010). In these cases, Cu(II) binding is weaker than Cu(I) binding as it was observed for CopM here. Surprisingly, CopM seems to bind only one Cu equivalent despite it contains a duplicated DUF305 domain (and each of this domains contain an HH motif and are rich in methionines) but this is similar to copper binding by PcoE. PcoE also present a high number of methionines and although it could bind several Cu(I) ions upon in vitro titration, it only retained one Cu(I) after purification of Cu-loaded PcoE by ion exchange (Zimmermann et al. 2012). In addition, our data also suggest that copper binding to CopM could also stimulate accumulation of the CopM processed form (and therefore exported; ppCopM), although both CopM forms were accumulated in response to copper (Figs.3, 4, 6, and 8). This was easier to visualize in the COPRM strain that expressed *copM* constitutively (although at low levels), in which ppCopM was clearly accumulated in response to copper (Fig.5). This suggests that on top of the transcriptional regulation of *copM*, there is a posttranscriptional level of regulation in CopM accumulation. Furthermore, CopM processing is not very efficient, as the unprocessed form of the protein is also accumulated. This could be related to the absence of a canonical transit peptide as the one present in CopM lacks a charge residue next to the cleavage site. On the other hand, CopM was also retained in columns charged with nickel (Fig.6), suggesting that CopM could have a role in nickel detoxification. However, we have not been able to detect any growth phenotypes in the presence of nickel in any of the *copM* mutants (unpubl. obs (Giner-Lamia et al. 2014) Furthermore, nickel is not able to induce *copMRS* and *copBAC* operons (Giner-Lamia et al. 2012, 2014) and therefore it is unlikely that this interaction could have any physiological effects but is probably mediated by the high number of histidine residues present in CopM, which are able to bind avidly nickel.

CopM has two DUF305 domains although very little is known about them beyond that they are characterized by a highly conserved two histidine H-H motif. According to this, the blastP analysis of the CopM protein sequence revealed several close homologs in cyanobacteria in which the H-H motif is strictly conserved. All of these proteins contain at least one DUF305 domain and many of them are located adjacent to copper-related genes. In the case of *Anabaena* sp. PCC 7120, we have found three ORFs (*all4988*, *all7594*, and *all7633*) that present a high identity to *copM*, and the transcriptional analysis showed that they are expressed in response to copper addition (Fig.9). All these data suggest that DUF305 motif could be involved in copper and other metal homeostasis in cyanobacteria.

Finally, we have also shown that the COPR mutant contained both higher intracellular copper and plastocyanin levels than the WT strain (Fig.8D). The higher plastocyanin accumulation is probably a mechanism to protect the cells from the damaging effects of higher copper content as it has been proposed that plastocyanin can function as a copper sink (Pesaresi et al. 2009). These two phenotypes were suppressed when *copM* was expressed in this mutant background (the COPRM strain), suggesting that CopM is able to prevent copper accumulation in this strain. As the COPRM strain does not express the *copBAC* operon (Fig. S6), this effect should be mediated directly by CopM. Because the DUF305 domains present in CopM are frequently found in secreted proteins according to the Pfam database, we explored the possibility that CopM could be exported to the extracellular media. In fact, we were able to detect CopM in the extracellular fractions and this represented ∼30% of the total CopM in the cultures (Fig.9). Furthermore, the export of copper-loaded CopM to the extracellular fraction could explain the reduced copper content of COPRM cells (when compared to COPR) which will otherwise require assuming an inhibitory role of CopM in copper import. However, to the best of our knowledge, it has not been previously described that a metallochaperone is partially located in the extracellular space and is able to reduce the whole cell copper content in bacteria. Several functions have been assigned to other periplasmic copper metallochaperones like functioning as copper-buffering systems (such as PcoE, CopC, or CopK; Jiang et al. 2005; Djoko et al. 2007; Zimmermann et al. 2012), as chaperones shuttling copper from and to transporters (like CusF or CopC; Djoko et al. 2007; Bagai et al. 2008; Mealman et al. 2012; Padilla-Benavides et al. 2014) or to donate copper to copper-containing proteins (like CueP, CopC, or SenC; Badrick et al. 2007; Blundell et al. 2013; Lohmeyer et al. 2012). Moreover, a direct role in copper export and/or copper binding outside the cells has not been described previously. It is possible that some of these other periplasmic copper-binding proteins could have a similar function to the described in this work for CopM, but their extracellular localization has been overlooked.

## Supporting Information

**Figure S1.** Analysis of periplasmic proteins in *Synechocystis*. SDS-PAGE of protein extracts from whole cells and cytosolic and periplasmic purified fractions of *Synechocystis* in the presence (+) or in the absence (−) of copper. WT cells were grown in BG11C-Cu medium to mid-log growth phase and exposed for 4 h to 1 *μ*mol/L of CuSO_4_. Five micrograms of total protein from each soluble extract was separated by 15% SDS-PAGE. Asterisks indicates protein bands that were excised and analyzed by PMF. Numbers correspond to Table S1. W, whole cells extracts; C, cytosolic extracts; P, periplasmic extracts; M, markers proteins. **Figure S2.** *copM* and *copB* transcripts analysis in response to copper. (A) Northern blot analysis of *copM* expression in response to copper addition in the WT strain. Total RNA was isolated from cells grown in BG11C-Cu medium to mid-log growth phase and exposed for 24 h to copper 1 *μ*mol/L. Samples were taken at the indicated times. The filter was hybridized with *copM* probe and subsequently stripped and rehybridized with an *rnpB* probe as a control. (B) Quantification of relative mRNA levels of *copM*, in response to copper addition. Radioactive signals of three independent experiments for each strain were quantified and averaged. RNA levels were normalized with the *rnpB* signal. Plots of relative mRNA levels versus time were drawn; error bars represent SE. (C) Northern blot analysis of *copM* and *copB* expression in response to different copper concentration in the WT strain. Total RNA was isolated from cells grown in BG11C-Cu medium to mid-log growth phase and exposed for 3 h to the indicated copper concentration. The filter was hybridized with *copM and copB* probes and subsequently stripped and rehybridized with an *rnpB* probe as a control. (D) Quantification of relative mRNA levels of *copM* and *copB*, in response to copper addition. Radioactive signals of three independent experiments for each strain were quantified and averaged. RNA levels were normalized with the *rnpB* signal. Plots of relative mRNA levels versus time were drawn; error bars represent SE. **Figure S3.** Construction and PCR analysis of the ΔΔ3 and ΔΔ3RS mutants strains. (A) Schematic representation of the *cop* genes in both genomic (*copMRS*) and pSYSX plasmid (*copMRS*) regions in the ΔΔ3 mutant strain. The *C.K1* and *Sp*Ω cassettes were inserted in each mutant strain at the indicated restriction sites. Crossed dashed lines show the recombination sites. (B) Schematic representation of the *glnN* gene region and the plasmid pCOPRS10+ used to generate ΔΔ3RS mutant strain. Crossed dashed lines show homolog recombination sites. Oligonucleotides used to verify the segregation of ΔΔ3RS mutant strain are shown in blue. (C) PCR analysis of genomic DNA isolated from merodiploid ΔΔ3RS and WT strains using oligonucleotides shown in B (Table S2). 1. PCR products from 50 to 51 primers. 2. PCR products from 50-NY2 primers. M, DNA ladder. **Figure S4.** Construction and PCR analysis of the WTM and COPRM mutants strains. (A) Schematic representation of the *glnN* gene region and the plasmid pCOPMR+ used to generate WTM and COPMR mutants strains. Crossed dashed lines show the recombination sites. Oligonucleotides used to verify the complete segregation of WTM and COPRM mutants strains are shown in blue. (B) PCR analysis of genomic DNA isolated from WTM, COPRM, and WT strains using oligonucleotides shown in A. M, DNA ladder. **Figure S5.** Analysis of *petE* expression in mutants strains affected in copper resistance. (A) Western blot analysis of plastocyanin levels after copper addition in WT, WTM, COPR, COPRM, and COPB strains. Cells were grown in BG11C-Cu medium to mid-log growth phase and exposed for 24 h to copper 1 *μ*mol/L. Five micrograms of total protein from soluble extracts were separated by 15% SDS-PAGE and subjected to western blot to detect plastocyanin (PC) and glutamine synthetase type I (GSI). (B) Northern blot analysis of *petE* expression for 24 h in response to copper addition in the WT strain. Total RNA was isolated from cells grown in BG11C-Cu medium to mid-log growth phase and exposed for 24 h to copper 1 *μ*mol/L. Samples were taken at the indicated times. The filter was hybridized with *petE* probe and subsequently stripped and rehybridized with an *rnpB* probe as a control. **Figure S6.** Transcriptional analysis of *copB* in WT and COPRM strains. Northern blot analysis of *copB* expression in response to copper addition in WT and COPRM strains. Total RNA was isolated from cells grown in BG11C-Cu medium to mid-log growth phase and exposed for 90 min to copper 1 *μ*mol/L. Samples were taken at the indicated times. The filter was hybridized with *copB* probe and subsequently stripped and rehybridized with an *rnpB* probe as a control.

**Table S1.** Proteins identified from the periplasmic extracts of *Synechocystis* sp. PCC 6803.

**Table S2.** Oligonucleotides used in this work.

## References

[b1] Achard ME, Tree JJ, Holden JA, Simpfendorfer KR, Wijburg OL, Strugnell RA (2010). The multi-copper-ion oxidase CueO of *Salmonella enterica* serovar Typhimurium is required for systemic virulence. Infect. Immun.

[b2] Arnesano F, Banci L, Bertini I, Mangani S, Thompsett AR (2003). A redox switch in CopC: an intriguing copper trafficking protein that binds copper(I) and copper(II) at different sites. Proc. Natl. Acad. Sci. USA.

[b3] Badarau A, Dennison C (2011). Thermodynamics of copper and zinc distribution in the cyanobacterium *Synechocystis* PCC 6803. Proc. Natl. Acad. Sci. USA.

[b4] Badrick AC, Hamilton AJ, Bernhardt PV, Jones CE, Kappler U, Jennings MP (2007). PrrC, a Sco homologue from *Rhodobacter sphaeroides*, possesses thiol-disulfide oxidoreductase activity. FEBS Lett.

[b5] Bagai I, Rensing C, Blackburn NJ, McEvoy MM (2008). Direct metal transfer between periplasmic proteins identifies a bacterial copper chaperone. Biochemistry.

[b6] Bersch B, Favier A, Schanda P, van Aelst S, Vallaeys T, Coves J (2008). Molecular structure and metal-binding properties of the periplasmic CopK protein expressed in *Cupriavidus metallidurans* CH34 during copper challenge. J. Mol. Biol.

[b7] Blundell KL, Wilson MT, Svistunenko DA, Vijgenboom E, Worrall JA (2013). Morphological development and cytochrome c oxidase activity in *Streptomyces lividans* are dependent on the action of a copper bound Sco protein. Open Biol.

[b8] Burkhead JL, Reynolds KA, Abdel-Ghany SE, Cohu CM, Pilon M (2009). Copper homeostasis. New Phytol.

[b9] Cai YP, Wolk CP (1990). Use of a conditionally lethal gene in *Anabaena* sp. strain PCC 7120 to select for double recombinants and to entrap insertion sequences. J. Bacteriol.

[b10] Chong LX, Ash MR, Maher MJ, Hinds MG, Xiao Z, Wedd AG (2009). Unprecedented binding cooperativity between Cu(I) and Cu(II) in the copper resistance protein CopK from *Cupriavidus metallidurans* CH34: implications from structural studies by NMR spectroscopy and X-ray crystallography. J. Am. Chem. Soc.

[b11] Davis AV, O'Halloran TV (2008). A place for thioether chemistry in cellular copper ion recognition and trafficking. Nat. Chem. Biol.

[b12] Depuydt M, Leonard SE, Vertommen D, Denoncin K, Morsomme P, Wahni K (2009). A periplasmic reducing system protects single cysteine residues from oxidation. Science.

[b13] Djoko KY, Xiao Z, Huffman DL, Wedd AG (2007). Conserved mechanism of copper binding and transfer. A comparison of the copper-resistance proteins PcoC from *Escherichia coli* and CopC from *Pseudomonas syringae*. Inorg. Chem.

[b14] Duran RV, Hervas M, De La Rosa MA, Navarro JA (2004). The efficient functioning of photosynthesis and respiration in *Synechocystis* sp. PCC 6803 strictly requires the presence of either cytochrome c6 or plastocyanin. J. Biol. Chem.

[b15] Espariz M, Checa SK, Audero ME, Pontel LB, Soncini FC (2007). Dissecting the *Salmonella* response to copper. Microbiology.

[b16] Finn RD, Bateman A, Clements J, Coggill P, Eberhardt RY, Eddy SR (2014). Pfam: the protein families database. Nucleic Acids Res.

[b17] Florencio FJ, Perez-Perez ME, Lopez-Maury L, Mata-Cabana A, Lindahl M (2006). The diversity and complexity of the cyanobacterial thioredoxin systems. Photosynth. Res.

[b18] Franke S, Grass G, Rensing C, Nies DH (2003). Molecular analysis of the copper-transporting efflux system CusCFBA of *Escherichia coli*. J. Bacteriol.

[b19] Fulda S, Huang F, Nilsson F, Hagemann M, Norling B (2000). Proteomics of *Synechocystis* sp. strain PCC 6803. Identification of periplasmic proteins in cells grown at low and high salt concentrations. Eur. J. Biochem.

[b20] Garcia-Dominguez M, Florencio FJ (1997). Nitrogen availability and electron transport control the expression of *glnB* gene (encoding PII protein) in the cyanobacterium *Synechocystis* sp. PCC 6803. Plant Mol. Biol.

[b21] Giner-Lamia J, Lopez-Maury L, Reyes JC, Florencio FJ (2012). The CopRS two-component system is responsible for resistance to copper in the cyanobacterium *Synechocystis* sp. PCC 6803. Plant Physiol.

[b22] Giner-Lamia J, Lopez-Maury L, Florencio FJ (2014). Global transcriptional profile of the copper responses in the cyanobacterium *Synechocystis* sp. PCC 6803. PLoS One.

[b23] Grass G, Rensing C (2001a). CueO is a multi-copper oxidase that confers copper tolerance in *Escherichia coli*. Biochem. Biophys. Res. Commun.

[b24] Grass G, Rensing C (2001b). Genes involved in copper homeostasis in *Escherichia coli*. J. Bacteriol.

[b25] Jiang J, Nadas IA, Kim MA, Franz KJ (2005). A Mets motif peptide found in copper transport proteins selectively binds Cu(I) with methionine-only coordination. Inorg. Chem.

[b26] Kim EH, Nies DH, McEvoy MM, Rensing C (2011). Switch or funnel: how RND-type transport systems control periplasmic metal homeostasis. J. Bacteriol.

[b27] Loftin IR, Franke S, Roberts SA, Weichsel A, Heroux A, Montfort WR (2005). A novel copper-binding fold for the periplasmic copper resistance protein CusF. Biochemistry.

[b28] Lohmeyer E, Schroder S, Pawlik G, Trasnea PI, Peters A, Daldal F (2012). The ScoI homologue SenC is a copper binding protein that interacts directly with the cbb(3)-type cytochrome oxidase in *Rhodobacter capsulatus*. Biochim. Biophys. Acta.

[b29] Macomber L, Imlay JA (2009). The iron-sulfur clusters of dehydratases are primary intracellular targets of copper toxicity. Proc. Natl. Acad. Sci. USA.

[b30] Mealman TD, Bagai I, Singh P, Goodlett DR, Rensing C, Zhou H (2011). Interactions between CusF and CusB identified by NMR spectroscopy and chemical cross-linking coupled to mass spectrometry. Biochemistry.

[b31] Mealman TD, Zhou M, Affandi T, Chacon KN, Aranguren ME, Blackburn NJ (2012). N-terminal region of CusB is sufficient for metal binding and metal transfer with the metallochaperone CusF. Biochemistry.

[b32] Merida A, Leurentop L, Candau P, Florencio FJ (1990). Purification and properties of glutamine synthetases from the cyanobacteria *Synechocystis* sp. strain PCC 6803 and *Calothrix* sp. strain PCC 7601. J. Bacteriol.

[b33] Mills SD, Jasalavich CA, Cooksey DA (1993). A two-component regulatory system required for copper-inducible expression of the copper resistance operon of *Pseudomonas syringae*. J. Bacteriol.

[b34] Monchy S, Benotmane MA, Wattiez R, van Aelst S, Auquier V, Borremans B (2006). Transcriptomic and proteomic analyses of the pMOL30-encoded copper resistance in *Cupriavidus metallidurans* strain CH34. Microbiology.

[b35] Munson GP, Lam DL, Outten FW, O'Halloran TV (2000). Identification of a copper-responsive two-component system on the chromosome of *Escherichia coli* K-12. J. Bacteriol.

[b36] Muro-Pastor AM, Herrero A, Flores E (2001). Nitrogen-regulated group 2 sigma factor from *Synechocystis* sp. strain PCC 6803 involved in survival under nitrogen stress. J. Bacteriol.

[b37] Nagarajan S, Sherman DM, Shaw I, Sherman LA (2012). Functions of the duplicated hik31 operons in central metabolism and responses to light, dark, and carbon sources in *Synechocystis* sp. strain PCC 6803. J. Bacteriol.

[b38] Navarro F, Florencio FJ (1996). The cyanobacterial thioredoxin gene is required for both photoautotrophic and heterotrophic growth. Plant Physiol.

[b39] Navarro F, Martin-Figueroa E, Florencio FJ (2000). Electron transport controls transcription of the thioredoxin gene (*trxA*) in the cyanobacterium *Synechocystis* sp. PCC 6803. Plant Mol. Biol.

[b40] Nicolaisen K, Hahn A, Valdebenito M, Moslavac S, Samborski A, Maldener I (2010). The interplay between siderophore secretion and coupled iron and copper transport in the heterocyst-forming cyanobacterium *Anabaena* sp. PCC 7120. Biochim. Biophys. Acta.

[b41] Osman D, Cavet JS (2008). Copper homeostasis in bacteria. Adv. Appl. Microbiol.

[b42] Osman D, Waldron KJ, Denton H, Taylor CM, Grant AJ, Mastroeni P (2010). Copper homeostasis in *Salmonella* is atypical and copper-CueP is a major periplasmic metal complex. J. Biol. Chem.

[b43] Osman D, Patterson CJ, Bailey K, Fisher K, Robinson NJ, Rigby SE (2013). The copper supply pathway to a Salmonella Cu, Zn-superoxide dismutase (SodCII) involves P(1B)-type ATPase copper efflux and periplasmic CueP. Mol. Microbiol.

[b44] Padilla-Benavides T, George Thompson AM, McEvoy MM, Arguello JM (2014). Mechanism of ATPase-mediated Cu+ export and delivery to periplasmic chaperones: the interaction of *Escherichia coli* CopA and CusF. J. Biol. Chem.

[b45] Pesaresi P, Scharfenberg M, Weigel M, Granlund I, Schroder WP, Finazzi G (2009). Mutants, overexpressors, and interactors of *Arabidopsis* plastocyanin isoforms: revised roles of plastocyanin in photosynthetic electron flow and thylakoid redox state. Mol. Plant.

[b46] Pontel LB, Soncini FC (2009). Alternative periplasmic copper-resistance mechanisms in Gram negative bacteria. Mol. Microbiol.

[b47] Puig S, Rees EM, Thiele DJ (2002). The ABCDs of periplasmic copper trafficking. Structure.

[b48] Rensing C, Grass G (2003). *Escherichia coli* mechanisms of copper homeostasis in a changing environment. FEMS Microbiol. Rev.

[b49] Rensing C, Fan B, Sharma R, Mitra B, Rosen BP (2000). CopA: an *Escherichia coli* Cu(I)-translocating P-type ATPase. Proc. Natl. Acad. Sci. USA.

[b50] Reyes JC, Florencio FJ (1994). A new type of glutamine synthetase in cyanobacteria: the protein encoded by the *glnN* gene supports nitrogen assimilation in *Synechocystis* sp. strain PCC 6803. J. Bacteriol.

[b51] Reyes JC, Muro-Pastor MI, Florencio FJ (1997). Transcription of glutamine synthetase genes (*glnA* and *glnN*) from the cyanobacterium *Synechocystis* sp. strain PCC 6803 is differently regulated in response to nitrogen availability. J. Bacteriol.

[b52] Rippka R, Deruelles J, Waterbury JB, Herman M, Stanier RY (1979). Generic assignment, strain histories and properties of pure cultures of Cyanobacteria. J. Gen. Microbiol.

[b53] Robinson NJ, Winge DR (2010). Copper metallochaperones. Annu. Rev. Biochem.

[b54] Sambrook J, Fritsch EF, Maniatis T (1989). Molecular cloning: a laboratory manual.

[b55] Sarret G, Favier A, Coves J, Hazemann JL, Mergeay M, Bersch B (2010). CopK from *Cupriavidus metallidurans* CH34 binds Cu(I) in a tetrathioether site: characterization by X-ray absorption and NMR spectroscopy. J. Am. Chem. Soc.

[b56] Sauer J, Dirmeier U, Forchhammer K (2000). The *Synechococcus* strain PCC 7942 *glnN* product (glutamine synthetase III) helps recovery from prolonged nitrogen chlorosis. J. Bacteriol.

[b57] Singh SK, Grass G, Rensing C, Montfort WR (2004). Cuprous oxidase activity of CueO from *Escherichia coli*. J. Bacteriol.

[b58] Su CC, Long F, Zimmermann MT, Rajashankar KR, Jernigan RL, Yu EW (2011). Crystal structure of the CusBA heavy-metal efflux complex of *Escherichia coli*. Nature.

[b59] Tottey S, Rich PR, Rondet SA, Robinson NJ (2001). Two Menkes-type ATPases supply copper for photosynthesis in *Synechocystis* PCC 6803. J. Biol. Chem.

[b60] Tottey S, Rondet SA, Borrelly GP, Robinson PJ, Rich PR, Robinson NJ (2002). A copper metallochaperone for photosynthesis and respiration reveals metal-specific targets, interaction with an importer, and alternative sites for copper acquisition. J. Biol. Chem.

[b61] Tottey S, Waldron KJ, Firbank SJ, Reale B, Bessant C, Sato K (2008). Protein-folding location can regulate manganese-binding versus copper- or zinc-binding. Nature.

[b62] Tottey S, Patterson CJ, Banci L, Bertini I, Felli IC, Pavelkova A (2012). Cyanobacterial metallochaperone inhibits deleterious side reactions of copper. Proc. Natl. Acad. Sci. USA.

[b101] Vioque A (1992). Analysis of the gene encoding the RNA subunit of ribonuclease P from cyanobacteria. Nucleic Acids Res.

[b63] Waldron KJ, Robinson NJ (2009). How do bacterial cells ensure that metalloproteins get the correct metal?. Nat. Rev. Microbiol.

[b64] Waldron KJ, Tottey S, Yanagisawa S, Dennison C, Robinson NJ (2007). A periplasmic iron-binding protein contributes toward inward copper supply. J. Biol. Chem.

[b65] Waldron KJ, Rutherford JC, Ford D, Robinson NJ (2009). Metalloproteins and metal sensing. Nature.

[b66] Wernimont AK, Huffman DL, Finney LA, Demeler B, O'Halloran TV, Rosenzweig AC (2003). Crystal structure and dimerization equilibria of PcoC, a methionine-rich copper resistance protein from *Escherichia coli*. J. Biol. Inorg. Chem.

[b67] Xiao Z, Wedd AG (2010). The challenges of determining metal-protein affinities. Nat. Prod. Rep.

[b68] Zhang XX, Rainey PB (2008). Regulation of copper homeostasis in *Pseudomonas fluorescens* SBW25. Environ. Microbiol.

[b69] Zimmermann M, Udagedara SR, Sze CM, Ryan TM, Howlett GJ, Xiao Z (2012). PcoE – a metal sponge expressed to the periplasm of copper resistance *Escherichia coli*. Implication of its function role in copper resistance. J. Inorg. Biochem.

