# Supplementary material for: CopM is a novel copper-binding protein involved in copper resistance in Synechocystis sp. PCC 6803
Source: Microbiologyopen. 2014 Dec 26;4(1):167–85. doi: 10.1002/mbo3.231 (PMC4335983; doi:10.1002/mbo3.231)
Supplement: Supplementary file 1 [file mbo30004-0167-sd1.pdf]

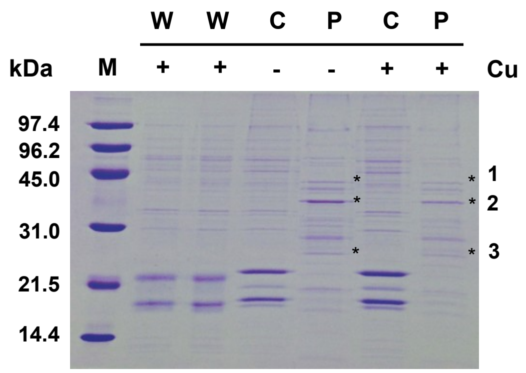

Figure S1. Analysis of periplasmic proteins in *Synechocystis*.
SDS-PAGE of protein extracts from whole cells and cytosolic and periplasmic purified fractions of *Synechocystis* in the presence (+) or in the absence (-) of copper. WT cells were grown in BG11C-Cu medium to mid-log growth phase and exposed for 4 h to 1 µM of $CuSO_4$. 5µg of total protein from each soluble extract was separated by 15% SDS-PAGE. Asterisks indicate proteins bands that were excised and analysed by PMF. Numbers correspond to Table S1. W, whole cells extracts, C, cytosolic extracts, P, periplasmic extracts. M, markers proteins.

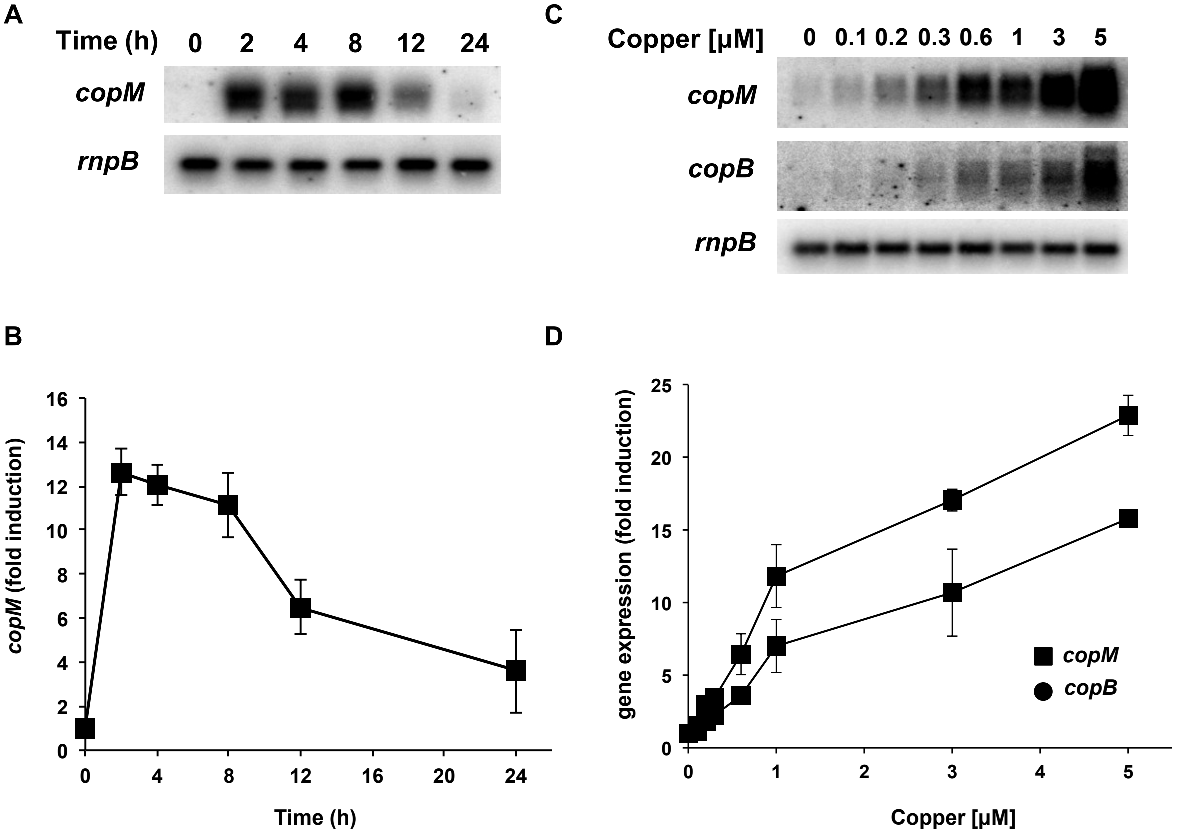

Figure S2. *copM* and *copB* transcripts analysis in response to copper.

A. Northern blot analysis of *copM* expression in response to copper addition in the WT strain. Total RNA was isolated from cells grown in BG11C-Cu medium to mid-log growth phase and exposed for 24h to copper 1 µM. Samples were taken at the indicated times. The filter was hybridized with *copM* probe and subsequently stripped and rehybridized with an *rnpB* probe as a control.

B. Quantification of relative mRNA levels of *copM,* in response to copper addition. Radioactive signals of three independent experiments for each strain were quantified and averaged. RNA levels were normalized with the *rnpB* signal. Plots of relative mRNA levels versus time were drawn; error bars represent SE.

C. Northern blot analysis of *copM* and *copB* expression in response to different copper concentration in the WT strain. Total RNA was isolated from cells grown in BG11C-Cu medium to mid-log growth phase and exposed for 3 h to the indicated copper concentration. The filter was hybridized with *copM and copB* probes and subsequently stripped and re-hybridized with an *rnpB* probe as a control.

D. Quantification of relative mRNA levels of *copM* and *copB,* in response to copper addition. Radioactive signals of three independent experiments for each strain were quantified and averaged. RNA levels were normalized with the *rnpB* signal. Plots of relative mRNA levels versus time were drawn; error bars represent SE .

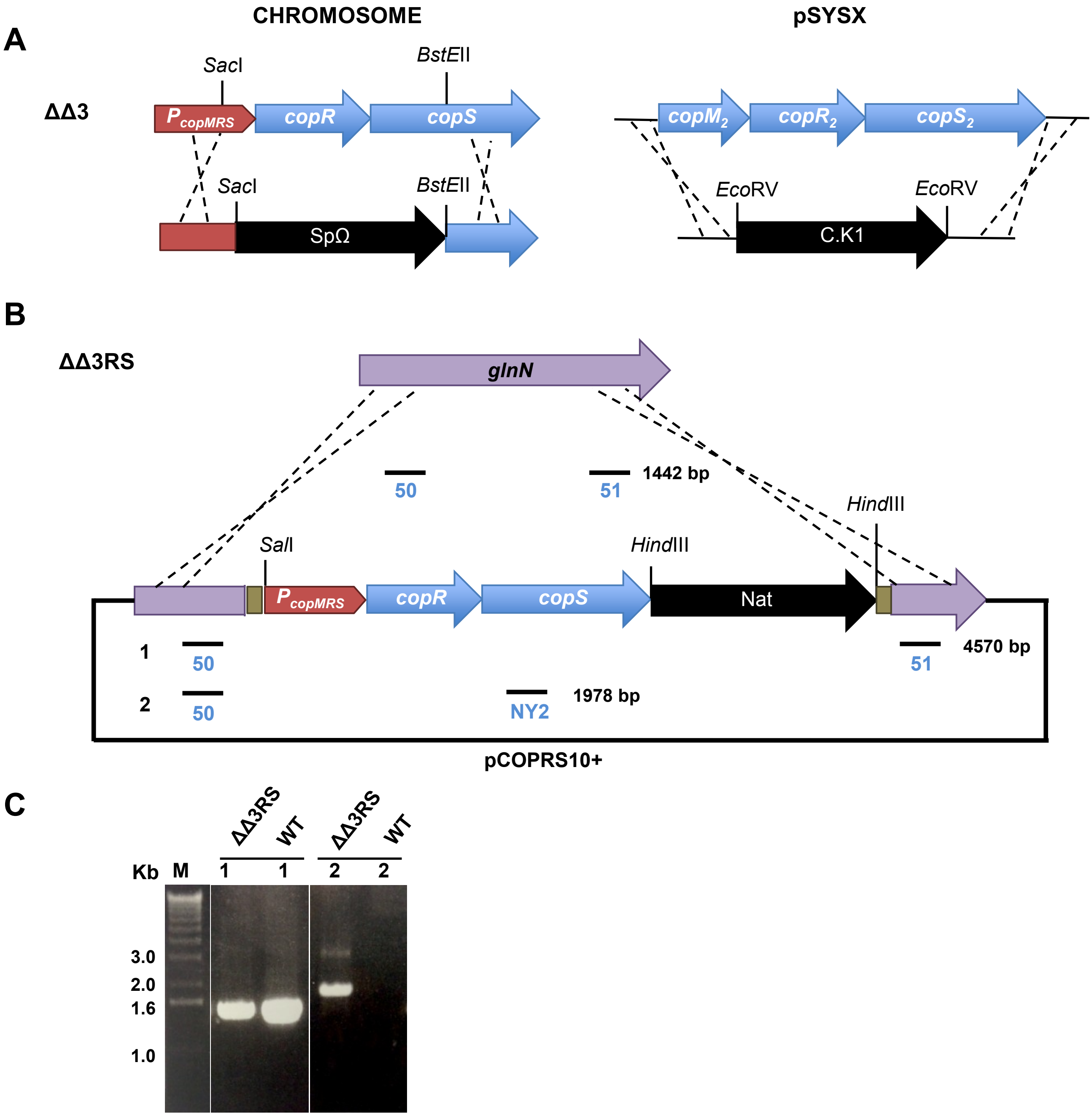

Figure S3. Construction and PCR analysis of the ΔΔ3 and ΔΔ3RS mutants strains.

A. Schematic representation of the *cop* genes in both genomic (*copMRS*) and pSYSX plasmid (*copMRS*) regions in the ΔΔ3 mutant strain. The *C.K1* and *Sp*Ω cassettes were inserted in each mutant strain at the indicated restriction sites. Crossed dashed lines show the recombination sites.

B. Schematic representation of the *glnN* gene region and the plasmid pCOPRS10+ used to generate ΔΔ3RS mutant strain. Crossed dashed lines show homolog recombination sites. Oligonucleotides used to verified the segregation of ΔΔ3RS mutant strain are shown in blue.

C. PCR analysis of genomic DNA isolated from merodiploid ΔΔ3RS and WT strains using oligonucleotides shown in B (table S2). 1. PCR products from 50-51 primers. 2. PCR products from 50-NY2 primers. M, DNA ladder.

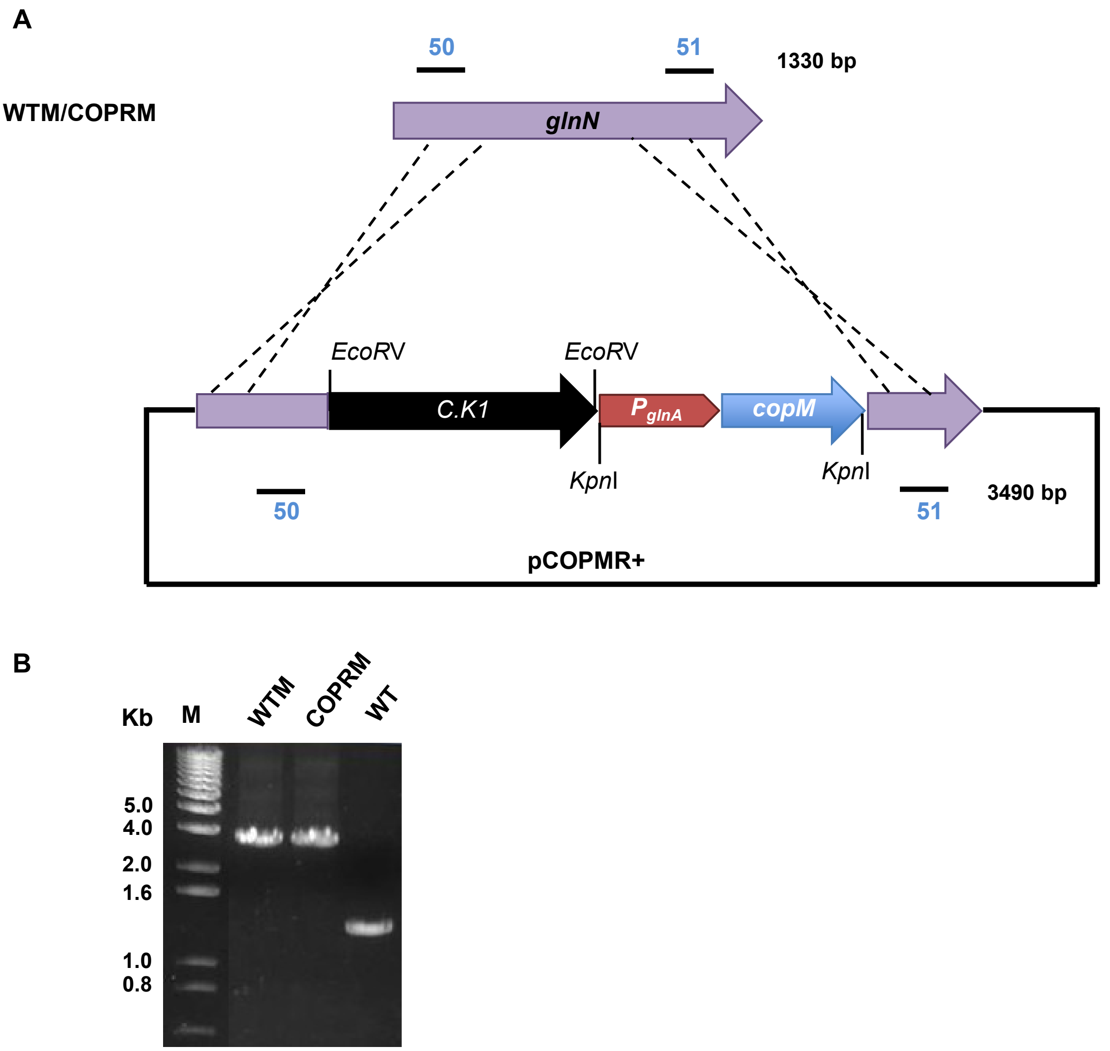

Figure S4. Construction and PCR analysis of the WTM and COPRM mutants strains.

A. Schematic representation of the *glnN* gene region and the plasmid pCOPMR+ used to generate WTM and COPMR mutants strains. Crossed dashed lines show the recombination sites. Oligonucleotides used to verified the complete segregation of WTM and COPRM mutants strains are shown in blue.

B. PCR analysis of genomic DNA isolated from WTM, COPRM and WT strains using oligonucleotides shown in A. M, DNA Ladder.

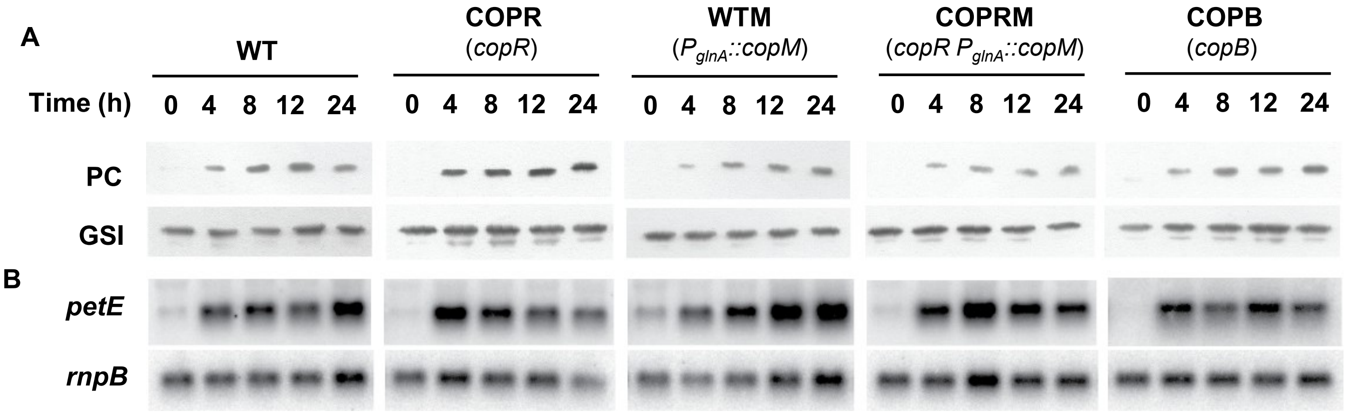

Figure S5. Analysis of *petE* expression in mutants strains affected in copper resistance.

A. Western blot analysis of plastocyanin levels after copper addition in wild-type (WT), WTM, COPR, COPRM and COPB strains. Cells were grown in BG11C-Cu medium to mid-log growth phase and exposed for 24h to copper 1 μM. 5 μg of total protein from soluble extracts were separated by 15 % SDS-PAGE and subjected to western blot to detect plastocyanin (PC) and glutamine synthetase type I (GSI).

B. Northern blot analysis of *petE* expression for 24h in response to copper addition in the wild-type strain. Total RNA was isolated from cells grown in BG11C-Cu medium to mid-log growth phase and exposed for 24h to copper 1 μM. Samples were taken at the indicated times. The filter was hybridized with *petE* probe and subsequently stripped and re-hybridized with an *rnpB* probe as a control.

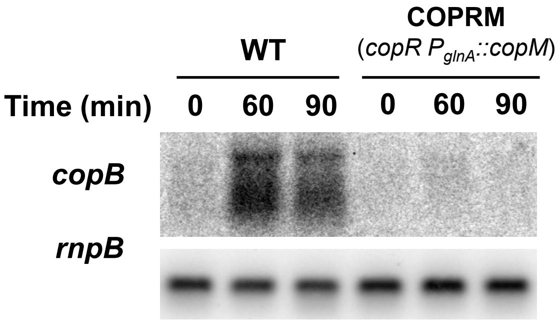

Figure S6. Transcriptional analysis of *copB* in WT and COPRM strains.
Northern blot analysis of *copB* expression in response to copper addition in WT and COPRM strains. Total RNA was isolated from cells grown in BG11C-Cu medium to mid-log growth phase and exposed for 90 min to copper 1 µM. Samples were taken at the indicated times. The filter was hybridized with *copB* probe and subsequently stripped and re-hybridized with an *rnpB* probe as a control.
