# Supplementary material for: CopM is a novel copper-binding protein involved in copper resistance in Synechocystis sp. PCC 6803
Source: Microbiologyopen. 2014 Dec 26;4(1):167–85. doi: 10.1002/mbo3.231 (PMC4335983; doi:10.1002/mbo3.231)
Supplement: Supplementary file 2 [file mbo30004-0167-sd2.pdf]

Table S1. Proteins identified from the periplasmic extracts of *Synechocystis* sp. PCC 6803.

Number[1)]	ORF	Gene	Gene Product	Mass (kDa)	Score
1	slr1751		Peirplasmic C-terminal peptidase	46.8	274
2	slr0513	*futA2*	Periplasmic iron-binding protein	38.2	310
3	slr0051	*ecaB*	Periplasmic beta-type carbonic anhidrase	27.9	88

1) Spot numbers in Fig. S1.
