# Supplementary material for: CopM is a novel copper-binding protein involved in copper resistance in Synechocystis sp. PCC 6803
Source: Microbiologyopen. 2014 Dec 26;4(1):167–85. doi: 10.1002/mbo3.231 (PMC4335983; doi:10.1002/mbo3.231)
Supplement: Supplementary file 3 [file mbo30004-0167-sd3.pdf]

Table S2. Oligonucleotides used in this work.

Name	Sequence
**PetEF**	ACAATCCTCGCTGGCCTTCT
**PetER**	CGACAACTTTGCCTACCATG
**copM1F**	AGCATTCCCATGGGTAATCAATTCTGGATAT
**copM1R**	CATCTCGAGTCACTGACCATACCAGTTTTGATA
**copBF**	AAGTCAATCGGCTCAGTCTG
**copBR**	GTAAACGACTTTGGGTTCTC
**copRF**	GTGGTAGTAACGCCAGATA
**copRR**	CTAAGCCATACACAGTTTC
**NY2**	GCCGTTGCTAATAATTGACCG
**glnNF**	CAATCAGTTCACCAAATCATCAAT
**glnNR**	ACATTTCCTGGTAGGTAGGCAGG
**pGSF**	GGTACCTACTTGCGGGTTGGGTATGGTC
**pGSR**	CAGAATTGATTACCCACTTTTTCTCCTTAGTGCAGTC
**pCOPMF**	GACTGCACTAAGGAGAAAAAGTGGGTAATCAATTCTGGATA
**pCOPMR**	GGTACCCATGCTTCCTCACTGACCATAC
**Gcop-1**	GGTATCACCTCGATAACCCTG
**Gcop-2**	CACCAGCAACAGTCTCATGGGAATGCTTACCTCCTA
**Gcop-3**	ATGAGACTGTTGCTGGTGGA
**Gcop-4**	ATTGACGGTACCGATATCCTGTTAGGTGAGA
**all4988F**	TTGTAGTGATGACATCCC
**all4988R**	TCCATGCTGTGATCATGA
**all7633F**	TTGTCATTGACTTTGGTAGCG
**all7633R**	ATGATTCATCATACCGTCAT
**all7594F**	TAACGCACAAGCACAGGTTT
**all7594R**	GCCATTTCCATTGCTTGAGT
**Ana_rnpBF**	GCAGACCAGTTAGCTTAACTG
**Ana_rnpBR**	CTTTACACGAGGGCGATTATC
